# A serious game for children with Attention Deficit Hyperactivity Disorder: Who benefits the most?

**DOI:** 10.1371/journal.pone.0193681

**Published:** 2018-03-15

**Authors:** Kim C. M. Bul, Lisa L. Doove, Ingmar H. A. Franken, Saskia Van der Oord, Pamela M. Kato, Athanasios Maras

**Affiliations:** 1 Yulius Academy, Yulius Mental Health Care Organization, Barendrecht, the Netherlands; 2 Department of Clinical Psychology, Erasmus University of Rotterdam, Rotterdam, the Netherlands; 3 Centre for Innovative Research across the Life Course, Coventry University, Coventry, United Kingdom; 4 Faculty of Psychology and Educational Sciences, KU Leuven, Leuven, Belgium; 5 Developmental Psychology, University of Amsterdam, Amsterdam, the Netherlands; 6 Cognitive Science Centre Amsterdam, University of Amsterdam, Amsterdam, the Netherlands; 7 Faculty of Engineering, Environment and Computing, Coventry University, Coventry, United Kingdom; TNO, NETHERLANDS

## Abstract

**Objective:**

The aim of the current study was to identify which subgroups of children with Attention Deficit Hyperactivity Disorder (ADHD) benefitted the most from playing a Serious Game (SG) intervention shown in a randomized trial to improve behavioral outcomes.

**Method:**

Pre-intervention characteristics [i.e., gender, age, intellectual level of functioning, medication use, computer experience, ADHD subtype, severity of inattention problems, severity of hyperactivity/impulsivity problems, comorbid Oppositional Defiant Disorder (ODD) and Conduct Disorder (CD) symptoms] were explored as potential moderators in a Virtual Twins (VT) analysis to identify subgroups for whom the SG intervention was most effective. Primary outcome measures were parent-reported time management, planning/organizing and cooperation skills.

**Results:**

Two subgroups were identified. Girls (n = 26) were identified as the subgroup that was most likely to show greater improvements in planning/organizing skills as compared to the estimated treatment effect of the total group of participants. Furthermore, among the boys, those (n = 47) with lower baseline levels of hyperactivity and higher levels of CD symptoms showed more improvements in their planning/organizing skills when they played the SG intervention as compared to the estimated treatment effect of the total group of participants.

**Conclusion:**

Using a VT analysis two subgroups of children with ADHD, girls, and boys with both higher levels of CD and lower levels of hyperactivity, were identified. These subgroups mostly benefit from playing the SG intervention developed to improve ADHD related behavioral problems. Our results imply that these subgroups have a higher chance of treatment success.

## Introduction

Attention Deficit-Hyperactivity Disorder (ADHD) is one of the most common neurodevelopmental disorders with a worldwide prevalence rate of 5% among children and adolescents [1]. In addition to the core symptoms of hyperactivity, impulsivity and inattention, children with ADHD show functional impairments in different areas of daily life such as planning their homework, estimating the time needed to complete an assignment, staying focused on tasks at hand and building and maintaining meaningful social relationships with their peers [2–4]. These problems have been shown to adversely impact the daily life functioning of children with ADHD as well as their academic performance in the long-term [5–7]. Recently, behavioral scientists and health care professionals have been exploring new ways to optimize the daily life functioning in children with ADHD by improving their engagement with treatment through the use of video games to support behavior change [8]. The integration of game elements into existing treatment procedures (also called gamification) has been shown to enhance motivation and treatment effectiveness [9–13].

The field of serious gaming is relatively new and therefore efforts are more focused on evaluating impacts on outcomes instead of identifying moderating variables that are linked to those outcomes. However, in addition to gathering evidence about the effectiveness of the Serious Game (SG) intervention on group-level as in Randomized Controlled Trials (RCTs) it is important to gather more information on an individual level and identify characteristics that predict favourable outcomes of the SG intervention [14–18]. This will enable healthcare professionals to optimize the match between individuals and their treatment and as such allocate children who are likely to benefit from these interventions to this intervention and offer alternative treatments for children for whom the intervention is less effective. This will support the development of personalized treatment allocation algorithms and promote behavior scientists and researchers to examine pre-defined individual characteristics that are predictive for favourable treatment outcomes. A mismatch between children’s pre-treatment characteristics and their treatment can elevate the risk of non-response to treatment, which means that emotional and financial burdens for children with neurodevelopmental disorders, their relatives and the wider community remain [17,18].

Most of the available literature on potential moderators in the treatment of children with ADHD is from the Multimodal Treatment of Attention Deficit Hyperactivity Disorder study [MTA-study; 19, 20], the most recent and largest treatment study in young patients with ADHD. The MTA study compared treatment as usual to behavioral intervention, medication or a combination of behavioral intervention and medication treatment. Amongst other moderation analyses, this study demonstrated that a certain pattern of comorbidity was associated with differential treatment responses. Specifically, they found that children with ADHD diagnosed with a single comorbid anxiety disorder (but NOT a comorbid CD) showed better treatment outcomes when receiving a behavioral intervention. While children with a double comorbidity (i.e., comorbid anxiety AND CD) benefitted the most from treatment combining medication and behavioral therapy [21, 22]. A more recent smaller scale study explored moderators of treatment response to a behavioral parent training combined with treatment as usual (TAU) as compared to stand-alone TAU [23, 24]. These results indicated that no or a single-type comorbidity [anxiety/depression or ODD / CD] is a significant moderator of treatment outcome demonstrating that behavioral parent training is more beneficial for these subgroups of children. Other possible moderating variables such as age, gender, ADHD severity, Intelligence Quotient (IQ) and medication use have been reported to be non-significant moderators of treatment outcomes in the MTA study [19–22, 25].

Other potential moderators relevant for the current study are gender and experience with computer gaming. A study by Chou and Tsai [26] demonstrated that boys spent more time playing computer games than girls and also enjoyed it more. Furthermore, previous research has shown that children with higher levels of game experience were more engaged with serious gaming [27]. Hence, this motivation, experience and engagement may enhance effectiveness of the Serious Game (SG) intervention [9, 28, 29].

As an adjunct to TAU for children with ADHD, an engaging and accessible serious game (called “Plan-It Commander”) aimed at improving functional outcomes in different areas of daily life such as time management, planning/organizing and cooperation skills was developed [14]. The effectiveness of “Plan-It Commander” was evaluated in a multisite open-label RCT (N = 170) and results showed that children with ADHD who played the SG intervention improved significantly more on the parent-rated primary outcome measure of time management and secondary outcomes measures of responsibility and working memory compared to children with ADHD who received TAU [15].

Due to the lack of moderation studies of serious gaming in children with ADHD, we based our hypotheses regarding the strength of specific moderators or the direction of effects on other studies examining moderator effects of treatment outcomes in ADHD [19–25]. In the current study, the role of 10 pre-intervention characteristics that were explored before in moderation studies [i.e., gender, age, IQ, medication use, computer experience, ADHD subtype, severity of inattention problems, severity of hyperactivity/impulsivity problems, comorbid ODD and CD symptoms] will be explored as possible moderators for the SG intervention on the primary outcomes [i.e. time management, planning/organization, cooperation] through a Virtual Twins (VT) analysis. This VT method explicitly aims at identifying subgroups that are likely to get a high benefit from the treatment as compared to the estimated treatment effect of the total group of participants [30]. VT has been successfully applied in the field of psychotherapy and pharmacogenetic research, see Doove and colleagues [30], and Hou and colleagues [31], respectively for some recent successful examples. In sum, the present study will use VT analysis to identify subgroups in which the effect of the SG intervention in improving several behavioural outcomes is considerably better than the effect of treatment as usual crossover group (TAUx).

## Materials and methods

### Participants

**Fig 1:**
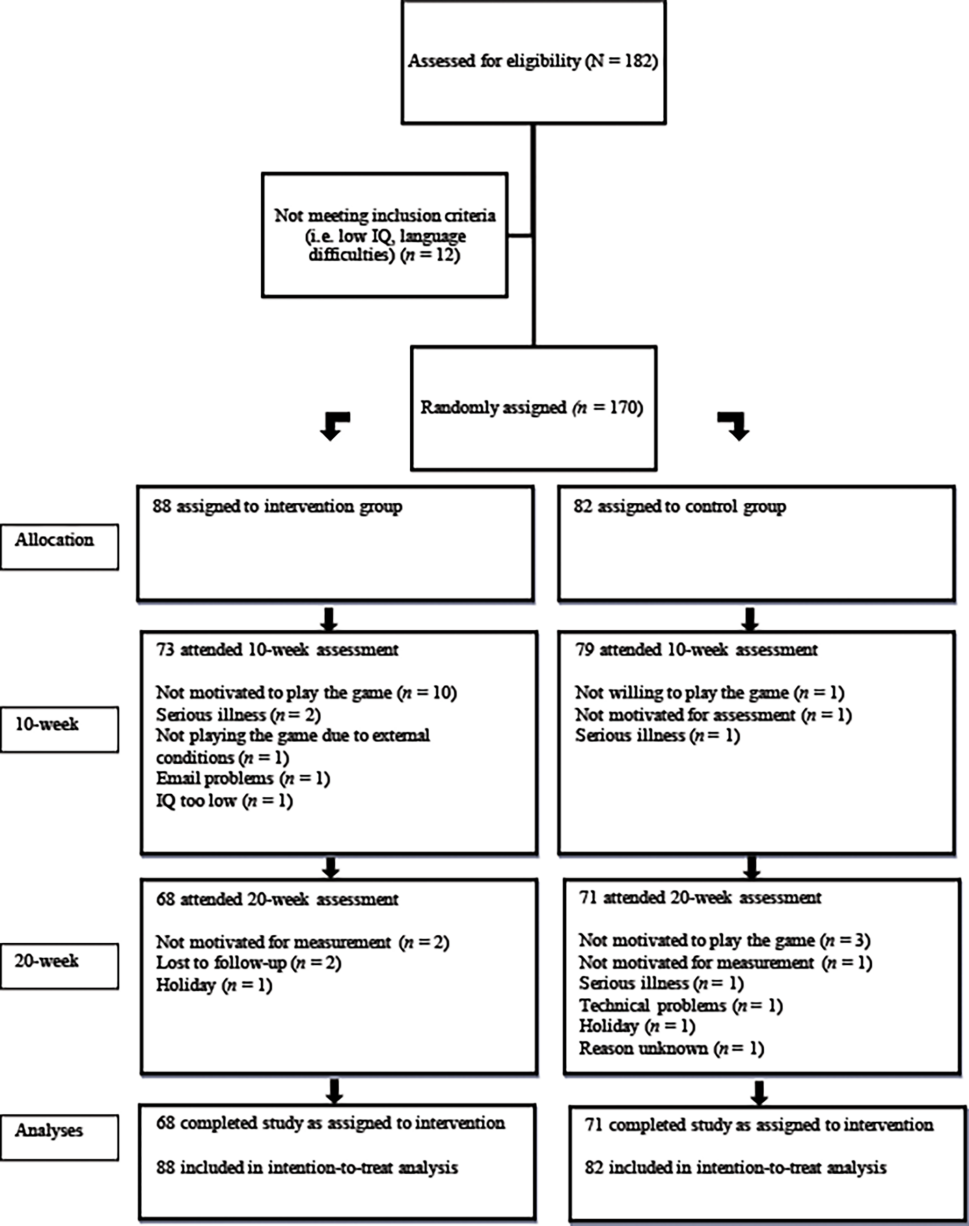
Study flow diagram.

This study used data from a 20-week multisite open-label RCT consisting of a Serious Game intervention (SG), developed to improve behavioral problems in children with ADHD, and a TAUx [15]. Across four sites in the Netherlands and Belgium, 170 participants were recruited and selected as part of the previous mentioned RCT, for more details on the study see [15]. Due to missing values on these outcome measures, the number of available cases for analysis was 143. Participants’ mean age was 9.90 years (SD = 1.26) at intake and they were all diagnosed with ADHD. The sample was 82% male and had an average IQ (M = 106; SD = 14.49). At baseline children had to be stable on pharmacological and/or psychological ADHD treatment for 8 weeks prior to baseline (determined by health care professionals on the basis of medication data and behavioral observation). Comorbidities common to ADHD like ODD were allowed. Participants with a clinical diagnosis of CD, autism spectrum disorder or acute psychiatric problems (i.e., substance abuse problems, depression, mania) requiring immediate or other medical treatment were excluded. Written informed consent was obtained from parents and 12-year-old children. All study procedures were approved by the Erasmus (Dutch) and Leuven (Belgian) Medical Ethical Committees (see S1 Fig). The trial is registered under ISRCTN62056259 (see http://www.controlled-trials.com/ISRCTN62056259). More details about the subject recruitment and retention can be obtained from Bul and colleagues [15], Fig 1 and the CONSORT checklist (see S2 Fig).

### Design

Participants randomly assigned to SG group received a serious game intervention in addition to treatment as usual for the first 10 weeks and then received treatment as usual for the next 10 weeks. Participants randomly assigned to the TAUx group received treatment as usual (medication in 92% of the cases) for the first 10 weeks and crossed over to the SG intervention in addition to treatment as usual for the subsequent 10 weeks. In this study, we compared outcomes between the SG intervention and TAUx conditions during the first ten weeks. Participants were instructed to play the serious game for one hour three times a week. Multi-informant (parent, teacher and self-report) measures were administered at baseline (T0), at 10 weeks (T1) and at 10-week follow-up (T2).

### Randomization and blinding

Randomization was carried out in a 1:1 ratio and based on a pre-specified computer in permuted blocks. Group assignment was performed online using the next available number on the randomization list corresponding to the site and gender of the participant. It was not possible to blind participants to their treatment allocation. After screening and baseline assessment, parents received an email with the notification to which group (SG vs. TAUx group) they were allocated. Although all efforts were made to keep the investigator blind during baseline assessments, full blinding of researchers at 10-weeks could not be guaranteed because participants were not prohibited from spontaneously talking about the game during the study period. Details about the design and allocation procedures are reported elsewhere [15].

### Intervention

The serious game is an online computer game (called “Plan-It Commander”), developed by health care professionals, game experts, researchers, parents and children with ADHD. It is designed to improve functional outcomes in daily life with a primary focus on time management, planning/organizing and cooperation skills among children with ADHD. Participants have their own password and identification to log on from their homes, in which they can access two game components: (a) a mission-guided game environment with minigames related to the learning goals of time management, planning/organizing and cooperation skills and, (b) a closed social community. Details of the game development and content are described in Bul and colleagues [14].

### Outcome measures

The primary outcome measures as in line with our previous analyses, were difference scores (i.e., change from baseline to post-test) of parent-reported time management, planning/organizing, and cooperation behavior skills [15]. These skills were primarily trained in the minigames (i.e., a small, isolated games within the larger game environment that integrate unique game elements offering tools to improve strategic behavior) of the SG intervention and the primary outcomes for the current study.

#### Time management

A time management questionnaire was used to measure children’s time management behavior (see S3 Fig). We used this time management questionnaire because at the time of our study no other standardized measures were available to measure this construct in young children. The questionnaire contains 11 items and addresses specific time management behaviors that are taught in the game. Parents were asked to rate the time management skills of their child on a 10-point Likert scale (ranging from true–not true). An example item is: “My child frequently uses his/her watch to check upon the time”. From a previous pilot study, it appeared that this questionnaire is reliable (α = .85; N = 42) and results from our RCT indicated comparable reliability in a larger sample of children (α = .83; N = 170) [15].

#### Planning/organizing skills

The parent-rated version of the Behaviour Rating Inventory of Executive Function [BRIEF; 32, 33] assesses executive functioning in home situations in children aged from 5–18 years. The questionnaire contains 86 items in eight non-overlapping clinical scales and two validity scales. The answers are scored on a 3-point Likert scale (never–sometimes—often). An item example is: “Forgets to hand in homework, even when it is completed”. For this study the subscale Plan/Organise, consisting of 12 items, was used in order to measure children’s planning/organizing skills. The BRIEF has good reliability and construct validity [34].

#### Cooperation skills

The Social Skills Rating System-parent version [SSRS; 35, 36] was used to measure social functioning in children from 8 to 12 years old. This questionnaire consists of four subscales (e.g., Cooperation, Responsibility, Assertiveness, Self-Control) of 10 items each. The answers are scored on a 3-point Likert scale (never–sometimes—often). The total scale consists of 38 items and has a possible range from 0 to 80. An item example is: ‘‘Helps you with household tasks without being asked”. For this study only the subscale Cooperation, consisting of 10 items, was used in order to measure children’s cooperation skills. The total scale score is reliable (α = .88; 36).

### Potential moderators

#### Socio-demographics

A questionnaire was constructed for this study for parents to report on their child’s background characteristics such as age (in years), gender, medication use (yes/no) and computer game experience. Parents provided information on how experienced their child was in computer gaming by assigning them to four categories (starter-amateur-experienced-expert).

#### Intelligence quotient

Children’s intellectual level of functioning (IQ) was assessed by two subtests (i.e., vocabulary and block design) of the Wechsler Intelligence Scale for Children III [WISC-III; 37, 38] This IQ estimation has satisfactory reliability (*r* = 0.91) and correlates highly with the full-scale IQ score [38]. This shortened IQ test was administered by trained research assistants.

#### ADHD diagnosis

The Kiddie-Schedule for Affective Disorders and Schizophrenia-Lifetime version [K-SADS; 39, 40] is a semi-structured interview designed to assess current and past episodes of psychopathology in children and adolescents according to the DSM-III-R and DSM-IV criteria. For this study the K-SADS was used to determine ADHD subtype. Parents were interviewed by trained research assistants. Given that participants were currently stable on pharmacological and/or psychological treatment for their ADHD core symptoms, parents answered questions about the past period in which ADHD symptoms were most severe.

#### Severity of ADHD symptoms

The Disruptive Behavior Disorders Rating Scale [DBDRS; 41, 42] provides a measure of DSM-IV disruptive behavior symptoms in children between 6 and 12 years old. It consists of 42 items and four subscales (that were used in this study): inattention (nine items), hyperactivity/ impulsivity (nine items), ODD (eight items), and CD symptoms (16 items). For this study severity scores of these subscales were used. Items are scored on a 4-point Likert scale, ranging from ‘‘not at all” to ‘‘very much”. The Dutch translation used showed has adequate reliability (α range = .88–.94) and construct validity [42, 43].

### Data analytic plan

As we had no a priori hypotheses on the potential treatment moderators that describe the subgroups of children for which the SG intervention strongly outperforms TAU, we could not use standard moderator analyses. Therefore, a factorial analysis of variance (ANOVA) with a first factor pertaining to treatment methods and a second one to subgroups [44], as well as regression analyses with suitable interaction terms being included in the regression model [45] could not address our research question. For such a situation in which no clear a priori hypotheses exist on the subgroups for which an intervention is especially effective, Virtual Twins (VT) [46] is a suitable method to use for generating hypotheses [45,47].

**Fig 2:**
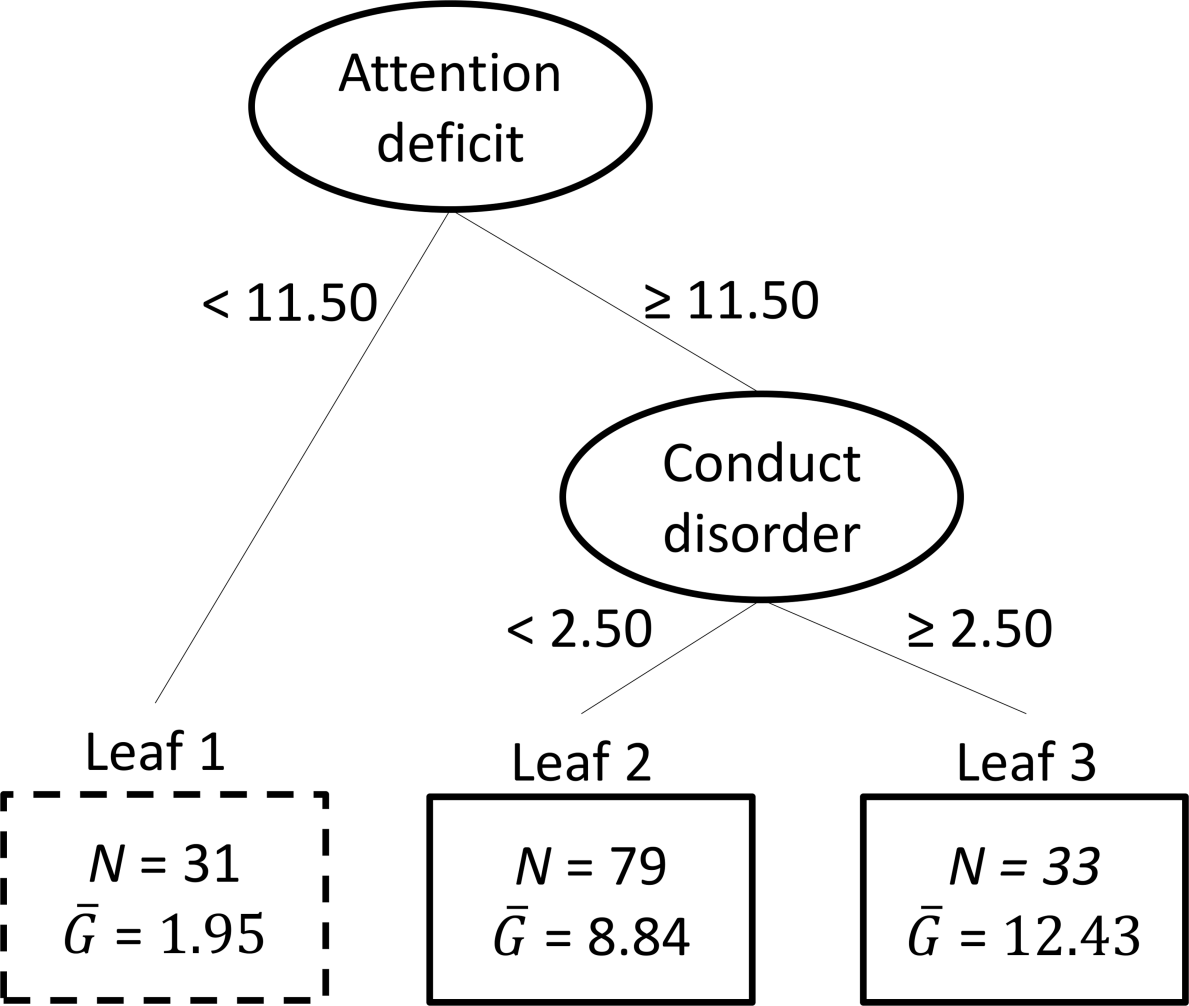
Results of the application of Virtual Twins with outcome time management. The ellipses in the figure represent the internal nodes containing the split variables, with below each ellipsis the corresponding split point. The upper ellipsis represents the root node corresponding to the complete group of children. The rectangles represent the leaves of the tree, that is, the final subgroups of children; each rectangle contains the sample size of the corresponding subgroup (N), and the mean estimated individual differential treatment effect ($\bar{G}$). The leaves in which the estimated individual differential treatment effect exceeds the threshold *c* = 8.18) are represented by solid rectangles. It should be noted that after post-processing of the results, it appears that there is insufficient evidence that the subgroups in these leaves have a significantly better outcome on time management skills compared to the total group of children. Subgroup estimated treatment effect was 8.07 (*d* = 0.56). Total group estimated treatment effect was 6.99 (*d* = 0.48).

The VT methodology is based on the concept of counterfactual or potential outcomes [48]. In VT analysis, for every person two potential outcomes can be defined; one for the SG intervention and one for TAUx. These two potential outcomes represent the outcomes that would be observed if the person in question were subject to the SG intervention or TAUx, respectively. As a first step, VT estimates for each person the two potential outcome values (also referred as virtual twins); the difference between these estimates represents for each person an estimate of that person’s individual differential treatment effect. In a second step of VT, this estimated individual differential treatment effect is entered as the criterion variable in a regression tree analysis [49], together with the available pre-intervention characteristics as predictors. Such a regression tree analysis implies that the total group of participants is repeatedly subdivided on the basis of binary splits of the pre-intervention characteristics into subgroups (i.e., leaves) that are increasingly homogeneous with respect to the criterion variable. The resulting series of splits can be represented by a tree structure like Fig 2 (which will further be discussed in the Result section). The union of the leaves of such a tree for which the average individual differential treatment effect exceeds some pre-specified threshold *c*, then constitutes the subgroup that is generated by the VT algorithm. Subsequently, an estimate of the enhanced treatment effect in the subgroup compared with the average treatment effect is determined. For this we will rely on the so-called re-substitution method as proposed by Foster and colleagues [46], where the difference between the estimated treatment effect in the subgroup and in the total group of persons is being calculated (based on observed rather than estimated potential outcome values).

In this study, we analyzed data on the three primary outcome measures with VT. Due to missing values on these outcome measures, the number of available cases for analysis was 143. As a threshold for determining which leaves of a fitted tree are part of the subgroup with enhanced treatment effect, we put *c* (i.e., the pre-specified threshold) equal to one standard error above the overall mean of estimated individual differential treatment effect. When VT outputs a subgroup with an enhanced treatment effect for one or more of the outcome measures, we will accompany the estimate of the enhanced treatment effect in the subgroup by confidence intervals as measure of reliability of the estimate (so-called percentile bootstrap confidence intervals) [50]. These confidence intervals give insight into whether there is sufficient evidence from the data that the subgroup found by VT has a significantly better outcome than the total group of participants.

VT analysis was performed using the ‘stats’ software package of R [51]. The R code for VT analysis is provided by Foster and colleagues [46]. Differences in sample characteristics between the two groups (SG vs. TAUx) were calculated using the independent t-test for continuous variables and the Chi-square test (or Fisher’s Exact Test when needed) for categorical variables. A significance level of < 0.05 was used with two-sided testing.

## Results

Presented in Table 1 are the pre-intervention characteristics and baseline scores of the three primary outcome measures for the participants enrolled in the current study. Mean scores and pre-intervention characteristics of the intervention and TAU only control groups did not differ significantly at baseline, Table 1.

**Table 1:** Sample characteristics.

	Total (N = 143)		SG (n = 64)		TAUx (n = 79)		Group Comparison	
**Pre-intervention characteristics**								
**Gender**								
**Boys**	117	(82%)	51	(80%)	66	(84%)	*χ*^2^ = 0.35,	*p* = .55
**Girls**	26	(18%)	13	(20%)	13	(16%)		
**Age in years**	9.90	(1.26)	10.00	(1.29)	9.82	(1.24)	*t =* -0.84,	*p* = .40
**Total IQ**^**a**^	105.93	(14.49)	104.52	(13.68)	107.08	(15.11)	*t =* 1.05,	*p* = .30
**ADHD subtypes (K-SADS)**								
**Combined**	105	(74%)	48	(75%)	57	(72%)		*p =* .09
**Inattentive**	32	(22%)	11	(17%)	21	(27%)		
**Hyperactive-Impulsive**	6	(4%)	5	(8%)	1	(1%)		
**ADHD symptoms**								
**Inattention**	16.08	(5.03)	15.30	(5.01)	16.72	(4.98)	*t =* 1.70,	*p* = .09
**Hyperactivity/Impulsivity**	13.86	(5.65)	14.44	(5.16)	13.39	(6.01)	*t =* -1.10,	*p* = .27
**Comorbidity**								
**ODD**	8.12	(4.92)	8.75	(5.26)	7.61	(4.60)	*t =* -1.39,	*p* = .17
**CD**	1.92	(2.53)	2.22	(2.65)	1.67	(2.42)	*t =* -1.29,	*p* = .20
**Game experience**								
**Starter/Amateur**	72	(50%)	32	(50%)	40	(51%)	*χ* ^2^ = 0.01,	*p* = .94
**Experienced/Expert**	71	(50%)	32	(50%)	39	(49%)		
**Medication use?**								
**Yes**	131	(92%)	58	(91%)	73	(92%)	*χ* ^2^ = 0.15,	*p* = .70
**No**	12	(8%)	6	(9%)	6	(8%)		
**Primary outcome measures at baseline**								
**Time management**	49.78	(16.38)	50.55	(17.57)	49.16	(15.44)	*t =* -0.50,	*p* = .62
**BRIEF (planning/organizing)**	20.90	(4.55)	21.53	(4.35)	20.38	(4.68)	*t =* -1.51,	*p* = .13
**SSRS (cooperation)**	8.74	(3.14)	8.73	(2.58)	8.75	(3.55)	*t =* 0.02,	*p* = .98

Independent sample t-tests were performed to calculate differences between continuous variables for which Mean +/- SD are presented. Chi-square test was used to calculate differences between categorical variables. Fisher’s Exact Tests were used in case of observations with expected value < 5.

^a^ IQ = Intelligence Quotient

^b^ * *p* < .05.

Based on our previous publication Bul and colleagues [15] it was indicated that children played on average 19 days in the mission-guided game and 11 days in the closed social community. With regard to playtime, participants played the mission-guided game for an average total duration of 13 hours and engaged with the closed social community for 54 minutes (on average). This suggests that not every child adhered to the recommended play frequency/time. Additional analyses including play time as a covariate did not affect previous findings reported in Bul and colleagues [15] with regard to its significance.

### Time management skills

Fig 2 displays the regression tree resulting from VT with time management as an outcome. As the overall mean of estimated individual differential treatment effect equalled 8.18, the threshold *c* was set to be equal to 8.18. This implied that the subgroup (n = 112) with enhanced treatment effect coincided with Leaves 2 and 3. The estimated treatment effect in this subgroup was 8.07 (*d* = 0.56), while the estimated marginal treatment effect in the total group of participants was equal to 6.99 (*d* = 0.48). Thus, the re-substitution estimate of the enhanced treatment effect in the subgroup compared with the average treatment effect (see Methods Section) equalled 1.08 (95% CI: -2.12, 3.80). As the 95% confidence interval around this estimate included 0, there was insufficient evidence that the subgroup identified using the VT method had a significantly better outcome on time management skills than the total group of participants. That is, none of the potential moderator variables in the VT analysis identified subgroups for whom the intervention increased time management skills.

### Planning/organizing skills (BRIEF)

**Fig 3:**
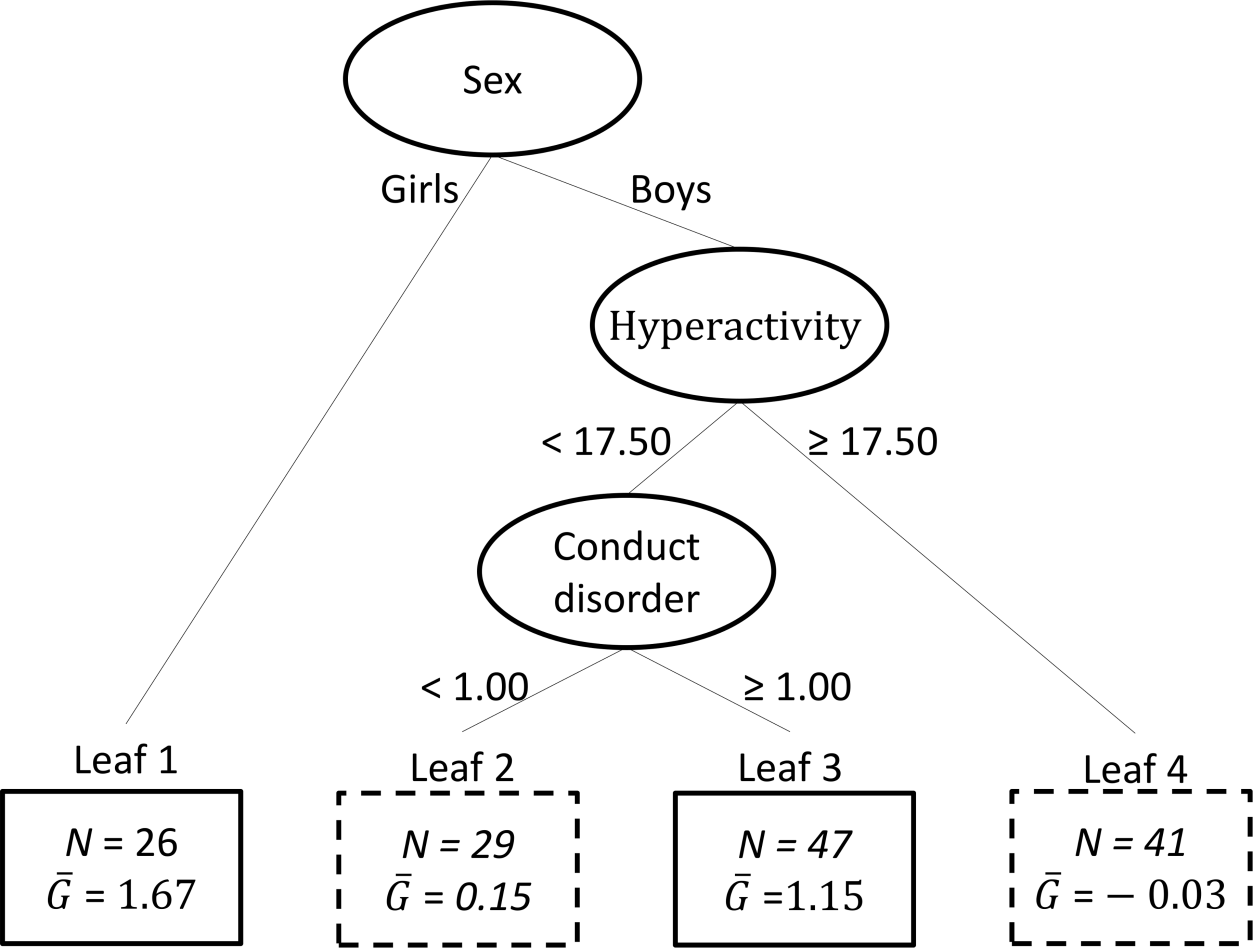
Results of the application of Virtual Twins with outcome Behaviour Rating Inventory of Executive Function (BRIEF). The ellipses in the figure represent the internal nodes containing the split variables, with below each ellipsis the corresponding split point. The upper ellipsis represents the root node corresponding to the complete group of children. The rectangles represent the leaves of the tree, that is, the final subgroups of children; each rectangle contains the sample size of the corresponding subgroup (N), and the mean estimated individual differential treatment effect ($\bar{G}$). The leaves in which the estimated individual differential treatment effect exceeds the threshold *c* = 0.67) are represented by solid rectangles. Post-processing of the results suggest that there is an indication that the subgroups in these leaves have a significantly better outcome on BRIEF compared to the total group of children. Subgroup estimated treatment effect was 2.17 (*d* = 0.65). Total group estimated treatment effect was 0.65 (*d* = 0.20).

Fig 3 displays the regression tree resulting from VT with planning/organizing skills as an outcome. The overall mean of the estimated individual differential treatment effect estimate equalled 0.67, which implied *c* = 0.67. This further implied that the subgroup (n = 73) with enhanced treatment effect coincided with Leaves 1 and 3. The estimated treatment effect in the induced subgroup was 2.17 (*d* = 0.65), while the estimated marginal treatment effect in the total group of participants was equal to 0.65 (*d* = 0.20). Thus, the re-substitution estimate of the enhanced treatment effect in the subgroup compared with the average treatment effect equalled 1.52 (95% CI: 0.43, 2.75). This suggested that the subgroup identified by the VT method had significantly better planning/organizing skills than the total group of participants. That is, two types of participants seemed to benefit from increased planning/organizing skills as a result of the intervention: 1) Girls (*n* = 26), and 2) Boys with both a lower score on hyperactivity (< 17.50) and a score on CD symptoms higher or equal to 1.00 (*n* = 47).

### Cooperation skills (SSRS)

**Fig 4:**
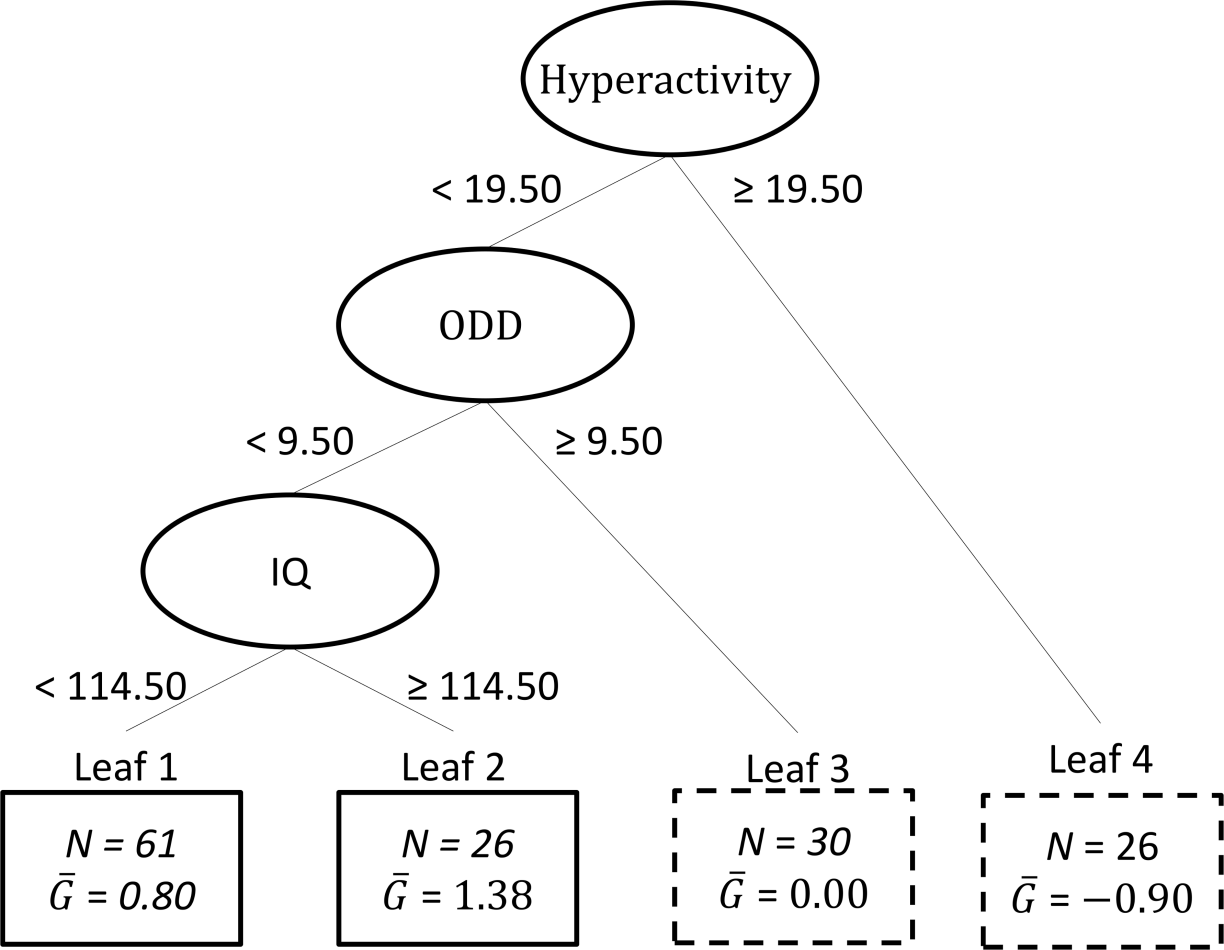
Results of the application of Virtual Twins with as outcome Social Skills Rating System (SSRS). The ellipses in the figure represent the internal nodes containing the split variables, with below each ellipsis the corresponding split point. The upper ellipsis represents the root node corresponding to the complete group of children. The rectangles represent the leaves of the tree, that is, the final subgroups of children; each rectangle contains the sample size of the corresponding subgroup (N), and the mean estimated individual differential treatment effect ($\bar{G}$). The leaves in which the estimated individual differential treatment effect exceeds the threshold *c* = 0.43) are represented by solid rectangles. It should be noted that after post-processing of the results, it appears that there is insufficient evidence that the subgroups in these leaves have a significantly better outcome on SSRS compared to the total group of children. Subgroup estimated treatment effect was 1.39 (*d* = 0.44). Total group estimated treatment effect was 0.69 (*d* = 0.23).

Fig 4 displays the regression tree resulting from VT on cooperation skills. The overall mean of the estimated individual differential treatment effect estimate equalled 0.43, which implied that *c* = 0.43. This further implied that the subgroup (n = 87) with enhanced treatment effect coincided with Leaves 1 and 2. The estimated treatment effect in the induced subgroup was 1.39 (*d* = 0.44), while the estimated marginal treatment effect in the total group of participants was equal to 0.69 (*d* = 0.23). The re-substitution estimate of the enhanced treatment effect in the subgroup compared with the average treatment effect thus equalled 0.70 (95% CI: -0.12, 1.58). The confidence interval around this estimate implied that there was insufficient evidence that the subgroup identified by the VT method had a significantly better outcome on cooperation skills compared with the total group of participants.

## Discussion

Although there is emerging research examining the effectiveness of interventions using game-based approaches and designs, there is still scant literature examining moderating variables. Results of a multisite open-label RCT indicated the superiority of a SG intervention on the parent-rated primary outcome measure of time management and secondary outcomes measures of responsibility and working memory [15]. We used the VT method to explore the role of several pre-intervention characteristics in predicting enhanced treatment effects on time management, planning/organizing and cooperation skills (included as primary outcome measures in the multisite open-label RCT). Although children appear to improve mainly on their time management skills (group-level), our moderator effects were particularly observed for the outcome variable of planning/organizing skills. We found that two groups benefitted on planning/organizing skills more from the SG intervention, compared with the estimated treatment effect of the total group: a) girls in general, and b) boys with both a lower score on hyperactivity/impulsivity symptoms and a higher score on CD symptoms. We found no effects of age, IQ, medication use, game experience and ADHD diagnosis on the three primary outcome measures. Our results imply that for these subgroups allocation to the SG intervention has a higher chance of treatment success.

In other moderation studies, for example the MTA study, gender was tested as a moderator, but was not associated with outcomes in any of the treatment conditions [19, 20, 25]. However, our results indicated that gender influences treatment success: in our study, girls benefitted more strongly from the intervention. Explaining this result in terms of lower levels of ADHD symptoms [52] or higher verbal IQ [53] among girls is not valid given that this was shown by the output of the VT analysis and therefore seen as unrelated to the enhanced intervention effects concerning plan/organize skills in the current subgroup. Therefore, other variables (e.g., motivation, parental characteristics) that were not measured in the current study might be at work and the underlying mechanism related to this favourable outcome of the SG intervention remains to be explored. As demonstrated by Van den Hoofdakker and colleagues [23, 24] parental characteristics can moderate treatment outcomes. Hence, our study, one of the first to examine gender as a moderator, contributes to the ADHD literature by demonstrating that game-based interventions are not equally effective for boys and girls.

In addition, we also found enhanced treatment effects for boys with both lower initial levels of hyperactivity/impulsivity and a higher CD symptom score. In our study, boys with scores falling in the normal to subclinical range of hyperactivity/impulsivity symptoms, who are therefore likely to be more stabilized on their TAU, benefitted the most from the intervention. In contrast, our finding with CD symptoms is more counterintuitive. CD symptoms are associated with aggressive/externalizing behavior, which suggests that they may interfere with treatment effectiveness. However, the CD symptoms score for our participants fell within the normal range (i.e., from 0 to 48), with a (sub)clinical cut off score at 5 depending on gender and age. This suggests that there were no serious clinical symptoms that could interfere with the intervention. Thus what mechanism causes this enhanced effect for this subgroup remains to be explored in further research.

In our previous study, we only found a marginal treatment effect of the SG intervention on participant’s planning/organizing skills (group-level). However, we did find a moderating effect on this outcome measure in the current study. This was surprising, and illustrates the value of examining moderator variables even if overall treatment effects are not present [15, 54].

### Clinical implications

Thus far, there has been a lack of studies examining moderating variables in the field of serious gaming within mental health care [55]. Apart from examining intervention effects on group level, it is important to examine moderator variables because individuals can show different treatment responses. The current study, one of the first to investigate the role of moderating variables, gives some indications for whom the intervention works best. These results could be used to direct more informed decisions regarding intervention allocation as it will enable healthcare professionals to optimize the match between individuals and their treatment. The results indicate that gender, levels of hyperactivity/impulsivity, and presence of CD symptoms should be taken into account when deciding upon treatment in clinical practice. Our results demonstrate that girls, and boys with both lower hyperactivity/impulsivity and a higher CD symptom score, benefitted the most from the intervention. In short, the SG intervention improves time management skills in children with ADHD (who already receive TAU) and enhanced treatment effects are present for specific subgroups on planning/organizing skills.

Our results are also important from a clinical perspective, as they may help stimulate personalized and targeted interventions. These results could contribute to a personalized clinical approach, indicating for whom an additional serious game treatment is most effective. In this regard, the DBDRS questionnaire [41–43], which was used in the current study to screen for ADHD and comorbid ODD and CD symptoms, could be used as a screener to support treatment indication. This questionnaire is a widely used and accepted instrument within the clinical field. Replication of the findings with larger samples is needed before reliable clinical implications about subgroups can be made [18]. These results should therefore be interpreted as more hypothesis generating and specific to the current sample.

### Limitations

There are also some limitations of this study that should be addressed in future work. First, although non-parametric approaches like VT have clear advantages over linear models [56, 57], in this study, the power to detect subgroups was relatively low. Despite this issue, we did find several significant moderators, to prevent ourselves from overfitting these data, we used a bootstrap-based procedure for estimating a measure of uncertainty. As compared to other RCTs, our sample size of children with ADHD was relatively large, but for the current method, even larger samples are needed to replicate these findings [58]. Second, although it is clinically important to determine long-term effects, we were not able to examine effects of moderation on the longer term [18], as these data were not available for the TAUx group. Third, although we included a broad range of moderators, we acknowledge that we did not investigate the potential role of other pre-intervention characteristics as possible moderators of treatment outcome. For example, we did not test the role of parental support, parental ADHD diagnosis, or the child’s motivation, all factors that have been demonstrated to be relevant by other studies [59, 60]. Finally, although our results are intriguing and when replicated relevant for clinical practice, we can only speculate why our subgroups show enhanced treatment effects. We hope that future research examining mediating factors will address this question as well.

## Conclusions

In conclusion, this study found two groups that benefitted most from SG intervention among a clinical sample of children with ADHD: girls in general, and boys with both a lower score on hyperactivity and a higher score on CD symptoms. These profiles should be considered when referring these patients to treatment. This work, together with future research on the mechanisms of the serious game, can begin to specify with greater precision the subgroups for whom treatment works best.

## Supporting information

S1 Fig Trial study protocol. (PDF)

S2 Fig CONSORT 2010 checklist. (DOC)

S3 Fig Time management questionnaire. (DOCX)
