# Supplementary material for: A serious game for children with Attention Deficit Hyperactivity Disorder: Who benefits the most?
Source: PLoS One. 2018 Mar 15;13(3):e0193681. doi: 10.1371/journal.pone.0193681 (PMC5854282; doi:10.1371/journal.pone.0193681)
Supplement: S1 Fig — (PDF) [file pone.0193681.s001.pdf]

The effectiveness of a serious game for children with ADHD: An open, randomized, controlled, multicenter trial.

Protocol amendment 1

**Sponsor Identifier**	CONCERTAATT4110
**Status:**	Final amendment 1
**Date:**	10 January 2013
**Prepared by:**	Janssen-Cilag BV in collaboration with Yulius Academy.
**GCP Compliance:**	This study will be conducted in compliance with Good Clinical Practice, and applicable regulatory requirements.

Confidentiality Statement

The information in this document contains trade secrets and commercial information that are privileged or confidential and may not be disclosed unless such disclosure is required by applicable law or regulations. In any event, persons to whom the information is disclosed must be informed that the information is privileged or confidential and may not be further disclosed by them. These restrictions on disclosure will apply equally to all future information supplied to you that is indicated as privileged or confidential.

INVESTIGATOR AGREEMENT

I have read this protocol and agree that it contains all necessary details for carrying out this study. I will conduct the study as outlined herein and will complete the study within the time designated.

I will provide copies of the protocol and all pertinent information to all individuals responsible to me who assist in the conduct of this study. I will discuss this material with them to ensure that they are fully informed regarding the study procedures, the conduct of the study, and the obligations of confidentiality.

Coordinating Investigator (where required):

Name (typed or printed): ________________

Institution and Address: ________________

Signature: ________________ Date: ________

(Day Month Year)

Principal (Site) Investigator:

Name (typed or printed): ________________

Institution and Address: ________________

Telephone Number: ________________

Signature: ________________ Date: ________

(Day Month Year)

Sponsor's Responsible Medical Officer:

Name (typed or printed): Helga van Oers

Institution: Janssen-Cilag BV

Signature: ________________ Date: ________

(Day Month Year)

Note: If the address or telephone number of the investigator changes during the course of the study, written notification will be provided by the investigator to the sponsor, and a protocol amendment will not be required.

CHANGE CONTROL

Comparison between Final version dated 28 Nov 2012 and Version Amendment 1 dated 8 January 2013	
Inclusion criteria (page 29)	Addition made to inclusion criterion 3 to clarify that stable treatment can include no treatment.
Screening measure Kiddie-SADS (page 37)	- Addition made to explain why diagnosis is made again during screening;<br>- Duration of measurement added.
Attachment K, page 89, 90	Change in choice option headers
Sample size (page 29, 30)	Sample size enlarged from 150 – 170 subjects
Statistical analyses (page 45)	Change from two sided test to Hochberg procedure
Primary outcome measures (page 43)	Change from multiple outcome measures to three outcome measures:<br>- BRIEF subscale planning and organization), total score of parents;<br>- Time management self constructed questionnaire, total score of parents;<br>- SSIS subscale cooperation, total score of parents.

TABLE OF CONTENTS

TITLE PAGE........1
INVESTIGATOR AGREEMENT........2
CHANGE CONTROL........3
LIST OF ABBREVIATIONS AND RELEVANT DEFINITIONS........6
SUMMARY........8
1. INTRODUCTION AND RATIONALE........11
2. OBJECTIVES........16
 2.1 Primary objective........16
 2.2 Primary research question........16
 2.3 Secondary objectives........16
 2.4 Secondary research questions........17
3. STUDY DESIGN........18
 3.1 Design........18
 3.2 Design rationale........19
 3.3 Duration/setting........19
 3.4 Measurements........20
 3.5 Burden of participants........26
4. STUDY POPULATION........29
 4.1 Sample........29
 4.2 Inclusion criteria........29
 4.3 Exclusion criteria........30
 4.4 Other conditions........30
 4.5 Sample size calculation........30
5. TREATMENT OF SUBJECTS........32
 5.1 Description of intervention........32
 5.2 Use of co-intervention........36
6. METHODS........37
 6.1 Study parameters/endpoints........37
  6.1.1 Screening measures........37
  6.1.2 Outcome measures........38
  6.1.3 Primary outcome measures........41
  6.1.4 Additional measures........41
 6.2 Randomisation, blinding and treatment allocation........42
 6.3 Signaling resistance........42
 6.4 Withdrawal of individual subjects........43
 6.5 Replacement of individual subjects after withdrawal........43
 6.6 Follow-up of subjects withdrawn from treatment........43
 6.7 Premature termination of the study........44
7. SAFETY REPORTING........45
8. STATISTICAL ANALYSES........46
9. ETHICAL CONSIDERATIONS........47
 9.1 Regulation statement........47

9.2 Recruitment and consent 47
9.3 Investigator Responsibilities 48
9.4 Independent Ethics Committee or Institutional Review Board 48
9.5 Objection by minors or incapacitated subjects (if applicable) 49
9.6 Benefits and risks assessment, group relatedness 49
9.7 Compensation for injury 50
10. ADMINISTRATIVE ASPECTS AND PUBLICATION 51
10.1 Handling and storage of data and documents 51
10.2 Amendments 51
10.3 Regulatory Approval/Notification 52
10.4 Required Prestudy Documentation 52
10.5 Subject Identification, Enrollment, and Screening Logs 52
10.6 Source Documentation 53
10.7 Data Quality Assurance/Quality Control 53
10.8 Record Retention 54
10.9 Monitoring 54
10.10 Study Termination 55
10.11 On-Site Audits 55
10.12 Annual progress report 55
10.13 End of study report 56
10.14 Public disclosure and publication policy 56
10.15 Registration of Clinical Studies and Disclosure of Results 57
11. STRUCTURED RISK ANALYSIS 58
11.1 Potential issues of concern 58
11.2 Synthesis 59
12. REFERENCES 60
13. ATTACHMENTS 67

LIST OF ABBREVIATIONS AND RELEVANT DEFINITIONS

**ABR**	**ABR form, General Assessment and Registration form, is the application form that is required for submission to the accredited Ethics Committee (In Dutch, ABR = Algemene Beoordeling en Registratie)**
**ADHD**	**Attention Deficit Hyperactivity Disorder**
**AE**	**Adverse Event**
**AR**	**Adverse Reaction**
**BRIEF**	**Behaviour Rating Inventory of Execution Function**
**CA**	**Competent Authority**
**CCMO**	**Central Committee on Research Involving Human Subjects; in Dutch: Centrale Commissie Mensgebonden Onderzoek**
**CV**	**Curriculum Vitae**
**eCRF**	**Electronic Case Report Form**
**EU**	**European Union**
**GCP**	**Good Clinical Practice**
**IB**	**Investigator's Brochure**
**IC(F)**	**Informed Consent (Form)**
**IRB/IEC**	**Independent Review Board/Independent Ethics Committee**
**K-SADS**	**Schedule for Affective Disorders and Schizofrenia for school - age children**
**ITT**	**Intention To Treat**
**ODD-CD**	**Oppositional Defiant Disorder and Conduct Disorder**
**(S)AE**	**(Serious) Adverse Event**
**SCVT**	**Social-Cognitive Skills Test (Sociale Cognitieve Vaardigheden Test)**
**SD**	**Standard Deviation**
**SES**	**Social Economic Status**
**SSIS-RS**	**Social Skills Improvement System Rating Scales**
**Sponsor**	**The sponsor is the party that commissions the organisation or performance of the research, for example a pharmaceutical company, academic hospital, scientific organisation or investigator. A party that provides funding for a study but does not commission it is not regarded as the sponsor, but referred to as a subsidising party.**
**TAU**	**Treatment As Usual**
**VvGK**	**Disruptive Behaviour Disorder Rating Scale (Vragenlijst voor Gedragsproblemen bij kinderen)**

**WAIS**	**Wechsles Adult Intelligence Scale**
**WISC-II**	**Wechsler Intelligence Scale for Children III**
**WMO**	**Medical Research Involving Human Subjects Act (in Dutch: Wet Medisch-wetenschappelijk Onderzoek met Mensen**

SUMMARY

Rationale: Psycho stimulants are recognized as the most effective treatment to reduce the core symptoms of Attention Deficit Hyperactivity Disorder (ADHD). Reducing the core symptoms of ADHD offers the possibility to stimulate and teach new behaviour. Additional interventions appear to be particularly valuable for this purpose. It is therefore important that a multimodal treatment also aims to reduce associated and co morbid problems, next to reducing the core symptoms of ADHD. It appears that these problems strongly predict the prognosis of ADHD and largely impact the daily functioning of the ADHD child and his/her family. Serious Gaming can possibly contribute to optimizing daily functioning of children with ADHD. The first results from the pilot study are promising.

Objective: The primary objective of this study is to examine the effectiveness of a serious game called HealSeeker for improving time management, planning and organisation and social behaviour skills of children with ADHD. The secondary objective of this research is to examine the effect of Healseeker on working memory and self-efficacy.

Study design: The design for this study consists of an open label randomised, controlled, multicenter trial. Onehundred and fifty participants will be randomly assigned to one of the two groups: an immediate and a delayed treatment group. Three measurement moments outside the game will take place. Furthermore, performances within the game will continuously be registred by a computer database.

Study population: This study will include a total of 170 children with ADHD (all subtypes), randomly selected among registered mental health care institutions, private psychology practices and paediatric practices across The Netherlands and Belgium. All participants need to be stable on ADHD treatment, pharmacological and/or psychological, for at least two months prior to start of the intervention. Participants must, when possible, continue this treatment throughout the intervention period. Further inclusion criteria are: 8 to 12 years of age, a minimum intelligence score of 80, a written informed consent signed by both parents or legal guardian (and the child, if he/she is 12 years of age), reasonable understanding of the Dutch language by one of the parents/legal guardians and child, access to a computer with internet and sound. Autism and conduct disorder as co-morbidities will be excluded.

Intervention (if applicable): The intervention in this study is a Serious Game called Healseeker. HealSeeker is an online computer game with a futuristic and adventurous character. Several missions and three minigames are embedded in the game. These minigames are related to three learning goals: time management, planning and organisation and social behaviour. A closed social community is included in which children can communicate with each other and ask each other for help through predefined messages. The

game is web-based, the children can play this game at home during the 10 weeks of the study. Children will be encouraged to play 3 times a week and each playing session will last a maximum of 45 minutes. Children can access the social community for a maximum of 20 minutes.

Main study parameters/endpoints: The main outcome variables are the time management skills, planning and organisation skills and social behaviour skills of the child. Secondary outcome variables are working memory and self-efficacy. The outcome variables will be evaluated by different questionnaires to be filled out by the child's parent/ legal guardian, the child's teacher and by tasks and a short questionnaire for the child.

Nature and extent of the burden and risks associated with participation: The extent of the burden and risks associated with participation in this study is limited. Children and parents/legal guardians have to invest time in filling out the questionnaires. It takes children approximately 215 minutes to fill out the questionnaires and perform neuropsychological tasks. For parent this takes approximately 245 to 275 minutes, for teachers this is 90 minutes. HealSeeker is played 3 times a week for 10 weeks at the child's home, therefore parents/legal guardians do not have to travel, which limits the extent of burden. The children spend a maximum of 135 minutes per week on the game. Apart from playing the game, children can play in the social community for a maximum of 60 minutes per week. Children diagnosed with ADHD are usually very motivated to play a computer game. The parents/legal guardians are not expected to be involved in this activity. Side effects to be expected are the side effects that can be expected from using a computer (like Repetitive Strain Injury (RSI)). Side effects of the game itself are not expected.

Benefit and group relatedness: This study provides children with ADHD the opportunity to join a non-medicinal intervention aimed at abating associated problems and elevating daily functioning. The game is web-based and can be played at the child's home during the study, therefore the intervention is accessible for a lot of children and parents/legal guardians.

1. INTRODUCTION AND RATIONALE

Prevalence of ADHD

Attention deficit/hyperactivity disorder (ADHD) is one of the most common developmental disorders, which manifests itself during childhood and often persists into adulthood (Wender, Wolf, & Wasserstein, 2001). The prevalence of ADHD is estimated to be around 3-6% for children and adolescents. Around two thirds of all children with ADHD have combined type, a quarter has mainly attention deficit and a minority (less than 10 percent) has predominantly hyperactive-impulsive type (Polancyk, Lima, Horta, Biedermam & Rohde, 2007). It is known that boys with ADHD often show more hyperactive and impulsive behaviour, while girls experience more problems with their concentration and attention (Nair, Ehimare, Beitman, Nair, & Lavin, 2006).

Associated problems of ADHD

Apart from the three core symptoms; attention deficit, hyperactivity and impulsivity; children with ADHD are generally characterised by a great number of associated problems (Barkley, 2006; Kats-Gold & Priel, 2009). Children with ADHD often lack self regulation, which causes them to experience difficulties in planning, organising, and time management (Barkley, 2006). Compared to normal developing children, children with ADHD encounter more difficulties in executing complex planning tasks, organising material needed for assignments, making a schedule to finish assignments on time, remembering task instructions and setting priorities (Abikoff et al., 2009). Furthermore, children with ADHD have difficulties to manipulate and reorganize visual spatial information (Martinussen, Hayden, Hogg-Johnson & Tannock, 2005). Due to an impaired working memory these children have trouble with remembering what (s)he was doing or what (s)he has to do to reach his or her current goal (Alloway, Gathercole & Pickering, 2006). Even though deficient social skills are not a part of the diagnostic criteria for ADHD, children with ADHD are often confronted with difficulties during interactions with peers (Mikami, 2010). They have the tendency to dominate a game, and they pay less attention to their peers during playing or other activities. Their interaction style is characterised by using orders and is therefore less reciprocal (Melnick & Hinshaw, 2000). Children with ADHD are aware of their socially inept behaviour, which causes them to be fearful in beginning new friendships and to derive little pleasure from interactions with peers (Kane, 2007). In sum, the associated problems these children experience lead to significant difficulties at school and in their social lives.

Pharmacological treatment of ADHD

Psycho stimulants are recognised as the most effective treatment of ADHD (MTA, 1999). They create a better balance of noradrenaline and dopamine in the prefrontal cortex of children with ADHD, which generally helps in decreasing the core symptoms of the disorder (MTA, 1999; Nationale Monitor Geestelijke Gezondheid, 2003; Schachter, Pham, King, Langford, & Moher, 2001). Even when the core symptoms of ADHD are managed through treatment with psychostimulants, one study (Abikoff et al., 2009) found that 61% of children on medication still experience difficulties with time management, planning and organising. These associated problems have been proven to predict the prognosis of ADHD, and greatly influence the impact of the disorder on family and daily life of the child (Vink & Van Wamel, 2007). This suggests that additional treatment is needed to help children manage these difficulties.

Non-pharmacological treatment as adjuvant therapy for ADHD

Additional interventions have proven to be of great value in modifying the behaviour of children with ADHD (Chronis, Jones, & Raggi, 2006; Pelham et al., 1993). There are some interventions available that try to reduce problems with planning, time management and social behaviour in children with ADHD. Abikoff et al. (2009) developed an intervention aimed to reduce these problems, but the effects of this intervention have not been published yet and the intervention is not available in the Netherlands. Furthermore, Kohlberg and Nadeau (2007) have published a self-help book - 'Opgeruimd leven met ADHD' - to improve planning and organising skills in adults with ADHD.

Several studies show that computerized working memory training programmes can improve attention deficit problems in children with ADHD. Klingberg, Forssberg and Westerberg (2002), for example, developed Cogmed training in order to improve working memory, which influences planning and organising skills. Twenty hours of training has proven to be effective in improving the working memory of children with ADHD, but the intervention is not specifically aimed at these children but on all people who have difficulties with working memory and the task is presented in a computer environment that does not promote engagement in the task over time. Melby-Lervåg and Hulme (2012) strongly doubt if the effects of working memory training can be generalized to daily life functioning. In addition, a recent study of Gibson et al. (2011) reviewed the Cogmed training and conclude that it can improve certain aspects of working memory but does not have an effect on the component of working memory that is particularly disturbed in children with ADHD. A study of Dovis, Van der Oord, Wiers, and Prins (2011) demonstrated that motivation can normalize working memory capacity and task persistence in children with ADHD. Gaming is a manner to improve motivation and thereby task persistence. Compared to the Cogmed training,

Healseeker is a game specially designed for children with ADHD and supposed to positively influence motivation and thereby optimizing performances.

Finally, there are several interventions that aim to improve social behaviour in children with ADHD, for example social skills training. This training helps children to develop age appropriate interpersonal skills. There is special attention for the sensitivity to the behaviour of self and others. However, several studies show that social skills training by itself is not enough to improve a broad spectrum of social competence or other related domains (Spence, 2003; Antshel & Remer, 2003). These kind of social interventions appear to be effective when combined with medication (Van der Oord, Prins, Oosterlaan & Emmelkamp, 2008). Although these interventions are still limited in its evidence and scope, it is important that multimodal treatments not only focus on the core symptoms, but also attempt to reduce the associated problems often found in children with ADHD. Serious gaming might possibly contribute to improving daily functioning of children with ADHD.

Serious gaming and ADHD

Serious games are also known as *social impact games* or *non-entertainment games,* meaning a game with a goal other than entertainment. A goal of a serious game could be for example education or training social skills. Gaming elements can be used to teach or convey a message in a fun and safe way. The entertaining character of games could be a motivation to use a serious game as a form of treatment. After all, games are challenging and fun to play, and it stimulates the player due to it's interactivity (Kranenburg, Slot, Staal, Leurdijk & Burgmeijer, 2006). Previous research has shown that interest and motivation of children with ADHD is positively influenced when learning tasks are offered in an attractive and interactive manner, such as a computer game (Emes, 1997; Tannock, 1997; Pfiffner, Barkley, Dupaul, 2006). Additionally, parents/legal guardians, teachers and clinicians report that while playing a computer game, children with ADHD have longer attention spans, are more concentrated and less impulsive (Barkley, 2006). Through giving frequent and direct reinforcement on a child's performance, optimising that performance will be stimulated.

Designing a serious game for children with ADHD is promising because children with ADHD appear to be motivated to play a computer game, and learn more this way, since they are more focused, more concentrated and less impulsive. Children with ADHD also tend to learn more effectively in an implicit way, though activities and experiences rather than studying books (Rosas et al., 2010). Because of this, it may be expected that children with ADHD could learn certain skills though an immersive and interactive serious game. A serious game which is tailored to meet the needs of these children could eventually lead to a decrease of associated problems in ADHD and complement the current treatment.

From a global search on the internet it appears that there are several ADHD games available online (see http://www.fupa.com /games/1/adhd.html). Unfortunately, these games have not been evaluated nor validated for the impact on outcomes. Some research has been done to examine the impact of games on attention span, relaxation and working memory in children with ADHD (Amon & Campbell, 2008; Pope & Bogart, 1996; Van der Oord, Ponsioen, Geurts, Brink, & Prins, 2012; Prins, Dovis, Ponsioen, Ten Brink, & Van der Oord, 2011). Although these computerized interventions are also focused on the target group of children with ADHD their aim and format is very different compared to HealSeeker. Pope and Bogart (1996) try to modify attention deficits through an extended attention span training. When the player's brain wave indicates that attention is weaning, the video game becomes more difficult so that attention from the player is re-initiated. Furthermore, the study of Amon and Campell (2008) is aimed to teach children with ADHD breathing and relaxation techniques through a game to diminish their disruptive behaviour patterns. Both studies are focused on reducing the core symptoms instead of associated problems of ADHD. Van der Oord and colleagues (2012) developed a neurcognitive training with three-dimensional game elements to attain the optimal level of performance on three executive functions (i.e. working memory, response inhibition and task switching). From the results it appeared that these skills are successfully trained in a timeframe of 12 hours. At the moment, this training is offered and examined in different target groups (i.e. autism and obesity). This implies that this training is not specifically designed for children with ADHD but for children who have difficulties with working memory.

Pilot Study Results

Janssen-Cilag BV (pharmaceutical company) initiated the idea to develop a serious game for children with ADHD in close collaboration with Yulius Mental Health Care Organisation and Ranj (serious game developer). The first prototype of HealSeeker has not been created from a point of view of already existing training programs, but instead an innovative gaming environment was created. HealSeeker is aimed at reducing associated problems in the area of time management, planning and social functioning and teaching children strategies to deal with situations in which these skills are needed and thereby improving self-management. HealSeeker is specifically designed for children from 8 to 12 years old. This young group of children has a good learning capacity which enables these children to develop at high speed and thereby feel competent. Training skills concerning planning, time management and social behaviour in children with ADHD at a young age, might prevent problems at school, at home and in relations during puberty.

From October 2011 till March 2012 a pilot study was conducted to test several game elements of the prototype HealSeeker for usability, and to examine the direction of possible

learning effects. In total, 42 children with ADHD were included in the study. Children were randomly assigned to two play conditions: a high frequent play condition (children were asked to play a minimum of eight times per two weeks) and a low frequent play condition (children were asked to play a maximum of three times per two weeks). Children played the game for eight weeks, divided in four periods of two weeks. Children were asked to play the game for a minimum of 30 minutes and a maximum of 45 minutes per game moment. Parents/legal guardians and children filled out several questionnaires at pre- and post-test. Data analyses were performed using complete data of 36 children. Children’s age ranged between 8 and 11 years with a mean age of 9.5 years. Twenty-six boys and 10 girls were included. From the results it appeared that parents/legal guardians were quite positive about the game. They rated HealSeeker with an average of 6.8 (range 6-10). Eighty-three percent of the parents/legal guardians would recommend this game to other parents/legal guardians of children with ADHD, once it is further developed. Ninety-four percent of the children liked to play HealSeeker. Preliminary results regarding the possible learning effects of HealSeeker provide an indication of therapeutic effects in the direction of improvement of time management and planning. Effects on social functioning could not be demonstrated. Intention to treat (ITT) analyses demonstrated similar results as the results stated above.

Based on these preliminary results, a final version of HealSeeker was developed. Specifically, the game was made more attractive and thereby thought to be more motivating and challenging for children. In addition, more learning situations were implicitly interwoven in game elements and the story line. A closed social community was included in which children could communicate with each other and ask each other for help through predefined messages.

2. OBJECTIVES

2.1 Primary objective

The primary objective of this study is to examine the effectiveness of HealSeeker for improving time management, planning and organisation and social behaviour skills of children with ADHD.

2.2 Primary research question

Does HealSeeker improve skills in the area of time management, planning and organisation and social behaviour skills of children with ADHD from 8 to 12 years of age? The expectation is that playing the HealSeeker game will lead to significant improvements in skills in the areas of time management, planning and organisation and social behaviour skills compared to a control group condition.

Van der Oord and colleagues (2012) investigated the effects of an executive function training program with game elements in children with ADHD. Results are encouraging and show a significant reduction in both ADHD behavioural symptoms and executive functioning problems. Moreover, recent evidence shows that serious games can be valuable in mental health care, for example to learn certain behavioural skills (Douma, 2008; Kato, 2010). A study about the effects of a serious game on willingness to help and role taking showed that interactive digital games can enhance empathic reaction to social issues (Peng, Lee, & Heeter, 2010). In addition, multiplayer online role-playing environments contain methodologies of role-play, modelling, instruction and reinforcement and by doing so social skills can be practised (Martin, 2009). Healseeker also uses elements of modelling, reinforcement and co-operation. Healseeker is an innovative program that does not purely train working memory but learn children strategies to cope with their difficulties in the area of time management, planning and social behaviour. Motivating and engaging children through a game is expected to optimize performances in these areas.

2.3 Secondary objectives

The secondary objective of this research is to examine the effects of Healseeker on working memory and self efficacy. In addition, it is examined whether intervention effects vary as a function of gender, age, ethnicity, SES, intelligence level, comorbidity, game experience, severity of psychopathology and satisfaction. The distribution of these baseline characteristics should be taken into account when answering this additional question.

2.4 Secondary research questions

- Does working memory serve as a mediating variable in the relationship between HealSeeker and outcome measures? The expectation is that playing the HealSeeker game will lead to significant improvements of working memory and that this will lead to improvements in outcome measures.

As mentioned earlier, working memory can be improved through computerized training programs. Improvements were also illustrated among other domains of executive functioning which supports the hypothesis that working memory is an intermediated factor in improving higher levels of executive functioning, for example planning and organisation skills (Beck, Hanson, Puffenberger, Benninger, & Benninger, 2010). Interestingly, Kofler and colleagues (2011) revealed a relationship between working memory deficits and social problems that was attenuated through its impact on ADHD symptoms. The core symptoms of ADHD, which can interfere with social behaviour, can be reduced by improving working memory capacity.

- Does self-efficacy serve as a mediating variable in the relationship between HealSeeker and outcome measures? The expectation is that playing the HealSeeker game will lead to significant improvements of self-efficacy and that this will lead to improvements in outcome measures.

Higher working memory capacity is positively related to self-enhancement (Schmeichel, 2010). There is evidence that games can improve self-efficacy of adolescents with cancer (Kato, Cole, Bradlyn, & Pollock, 2009). Additionally, it appears that neurofeedback and computerized attention tasks can positively influence self-management abilities of children with ADHD (Gevensleben et al. 2010). When children have higher levels of confidence in their skills this will eventually lead more effective learning (Bandura, 2006).

For more information about outcome variables see paragraph 6.1.

3. STUDY DESIGN

3.1 Design

The design for this study consists of an open label randomised, controlled trial. All efforts are made to make the investigator blind. Participants, their parents and teachers are not blind to treatment allocation. To determine the effectiveness of HealSeeker for improving time management, planning and organisation and social behaviour skills, 170 participants are randomly assigned to one of the two groups: an immediate and delayed treatment group (see Figure 1). The distribution of subjects between the immediate and delayed treatment group is 1:1.

Figure 1. Flowchart

Group 1: Immediate group

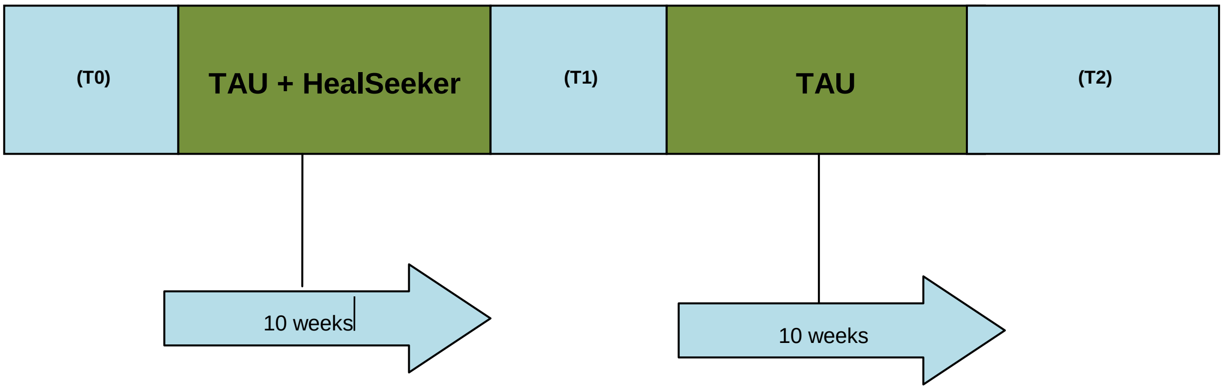

Before the intervention period pre-test measurements (T0) will take place. After the intervention period post-test measurements (T1) will take place. After 10 weeks a follow-up measurement (T2) will take place to measure long-term effects. Treatment As Usual (TAU) will be continued for all subjects in all periods.

Group 2: Delayed group

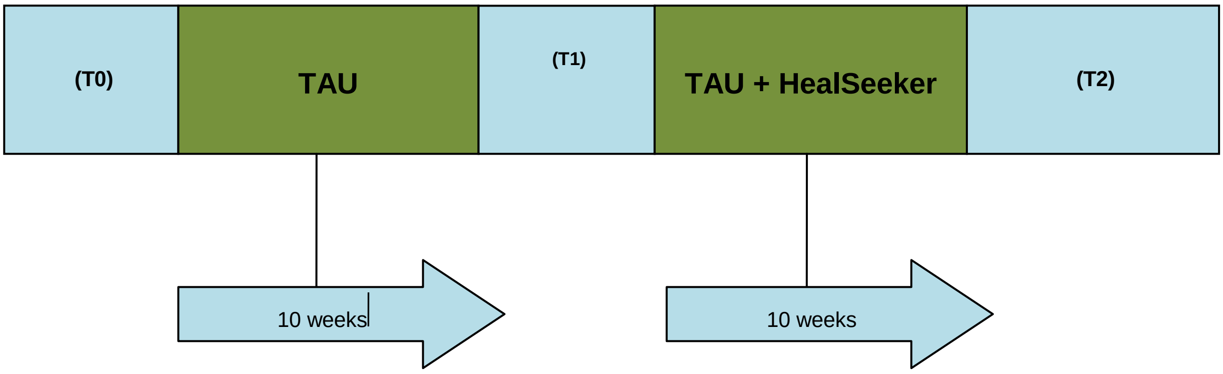

At start pre-test measurements (T0) will take place. After 10 weeks post-test measurements (T1) will take place. These post-test measurements will be the baseline measurements before entering the intervention condition. After 10 weeks final measurements (T2) will take place. Treatment As Usual (TAU) will be continued for all subjects in all periods.

3.2 Design rationale

In the absence of an appropriate control game a wait list control design was chosen. This design allows to assess the effect of the intervention as add-on to standard care while controlling for potential external confounders. Allocation to treatment group will be done trough an automated randomisation process to avoid introduction of bias in treatment allocation. Even though the trial will be open-label, efforts will be made to keep the assessor blinded to the treatment allocation during the trial to avoid bias in the assessment of subject outcome.

3.3 Duration/setting

As soon as the trial has been approved by the Independent Review Board/ Independent Ethics Committee (IEC/IRB) according to local standards screening visits can take place. Screening and measurements (T0, T1 and T2) will be taken at the research sites in the morning with a maximum time slot of 14.00 am. It is important to test children in the morning because they appear to be more concentrated especially if they are on medication. In addition, this will keep the variation between children low. Between the T0 and T1 visit and between the T1 and T2 visit the period should be 10 weeks in length (with a recommended visit window of + 7 days). For study visits that are unable to be held within the recommended hours and visit window time frame, the visit should be conducted as closely as possible to the protocol-specified study visit timing, but the period in between visits cannot be shorter than 10 weeks. All subsequent visits should be scheduled based on the date of the T0 measurement, not the date of the rescheduled visit. The questionnaires for the parent/legal guardian will be available on laptop, and offered in a quiet surrounding. The investigator will emphasize that it is important that the questionnaires are filled out by the same parent on all measurement moments. The questionnaires and tasks for the child will be performed in a separate quiet room. Tasks for the child will be taken at the same time of day on all three measurements moments (T0, T1 and T2). The questionnaires for the teachers are presented online, during three measurements occasions (T0, T1 and T2). They can fill these out at work or at home. The HealSeeker game is web based, and therefore accessible at home using a unique log-in name and password.

3.4 Measurements

To measure the effects of HealSeeker, this study uses both questionnaires and neuropsychological tasks administered to the child, and several questionnaires and checklists administered to their parents/legal guardians and teachers. Whilst playing HealSeeker, useful parameters for planning, time management and social skills will be saved in a computer database attached to the HealSeeker. First of all screening of subjects will take place, where informed consent will be signed and will be evaluated whether all in/exclusion criteria are fulfilled. If possible screening and T0 measurement will take place at the same visit. Measurements will take place at three different time intervals (T0, T1 and T2). More information concerning the measuring instruments used, and primary and secondary outcome variables is provided in paragraph 6.1: Study parameters/endpoints. The time and event schedule is illustrated in Table 1.

Table 1. *Time and event schedule.*

	Week 0	Week 0	Week 10	Week 20
**Study Procedure**	Screening / T0 Measurements prior to randomization	T0 measurements after randomization	T1 measurements	T2 measurements
Screening / Administrative (Investigator / site personnel)				
Informed consent/assent (ICF) [a]	X			
Demographics	X			
Diagnostic interview (DSM-IV diagnosis ADHD)	X			
Intelligence WISC-III-NL (only if necessary)[b]	X			
Inclusion/exclusion criteria	X			
Randomize[c]		X		
Adverse Events		X	X	X
Concomitant therapy regarding ADHD		X	X	X
Parents				
General questionnaire		X		
Severity of symptoms (VvGK) (Parents / Legal Guardian)		X		
Time management (self constructed questionnaire and It's About Time questionnaire)		X	X	X
BRIEF (planning, organizing, working memory subscale)		X	X	X
Social skills (subscale SSIS-RS)		X	X	X
Game experience of the child		X		
Satisfaction HealSeeker (self constructed questionnaire)[de]			X [d]	X [e]
Children				
Planning (neuropsychological task)		X[f]	X	X
Social skills (SCVT)		X[f]	X	X
Working memory (neuropsychological task)		X[f]	X	X
Self-efficacy (self constructed questionnaire)		X[f]	X	X
Satisfaction HealSeaker (self constructed questionnaire) [de]			X[d]	X[e]
Teacher				
Time management (self constructed questionnaire)		X	X	X
Social skills (subscale social skills rating scale)		X	X	X
BRIEF (planning, organizing, working memory subscale)		X	X	X

[a]Informed assent applicable for children of 12 years old.
[b]Only necessary if no intelligence test results are available of 2 years prior to screening.
[c]Randomize after all in/exclusion criteria fulfilled and study procedures above.
and VvGK (Parents / Legal Guardian) are performed.
[d]Satisfaction HealSeeker at T1 only for parents and children who played HealSeeker in the first 10 weeks (immediate treatment group).
[e] Satisfaction HealSeeker at T2 only for parents and children who played HealSeeker in the second 10 weeks (delayed treatment group).
[f] Tasks of the child at T0 may take place prior to randomization, when parent is interviewed for diagnosis.

Screening/T0 measurements prior to randomization

Potential subjects will be seen at a screening visit (T0 visit), at which informed consent will be obtained from their both parents/legal guardians. From children of 12 years informed consent will be obtained. To confirm the inclusion and exclusion criteria and to gather general information about the child the following measurements will be done during the screening phase:

- Intelligence *(*only if necessary, using WISC-III-NL)
- DSM-IV diagnosis ADHD and co morbidity (using K-SADS and parent interview)

T0 Measurement after randomization/T0 Visit

After screening measurements, but at the same visit, children who meet the enrolment criteria will directly continue with T0 measurement (if possible). HealSeeker will be explained to children and their parents/legal guardians. To assess the level of skills at baseline measurement, the following measurements will be done:

Parent/legal guardian:

- General questionnaire
- Severity of problems (using VvGK)
- Time management (using a self-constructed questionnaire and the It's about time questionnaire)
- Planning (using the planning and organising subscale of the BRIEF)
- Social skills (using a subscale of the SSIS-RS)
- Working memory (using the working memory subscale of the BRIEF)
- Game experience of the child (using a self-constructed questionnaire)

Children:

- Planning (using a neuropsychological task; tower test)
- Social skills (using SCVT)
- Working memory (using a neuropsychological task; chessboard task)
- Self-efficacy (using a self-constructed questionnaire)

Teacher:

- Time management (using a self-constructed questionnaire)
- Planning (using the planning and organising subscale of the BRIEF)
- Social skills (using a subscale of the SSIS-RS)
- Working memory (using the working memory subscale of the BRIEF)

The teacher will be approached via a electronic news letter and asked to fill out some questionnaires about the participating child. They can access the questionnaires online through a link provided in an email. After T0 measurements children will be randomized in a 1:1 to immediate or delayed treatment group.

Course of the intervention

To optimise the monitoring and documentation of the progress, duration, frequency and drop-out of the intervention, a database attached to HealSeeker will be used. During the intervention period all relevant parameters will be stored here (see paragraph 6.1: Study parameters/endpoints). During the intervention period parents/legal guardians can indicate on a schedule how much effort they had to make to motivate their child per game moment (see attachment M).

T1 Measurement/T1 visit

To assess the level of skills one week after the end of the intervention, as well as the satisfaction of the intervention, the following measurements will be done:

Parent/legal guardian:

- Time management (using a self-constructed questionnaire and the It's about time questionnaire)
- Planning (using the planning and organising subscale of the BRIEF)
- Social skills (using a subscale of the SSIS-RS)
- Working memory (using the working memory subscale of the BRIEF)
- Satisfaction regarding HealSeeker (using a self-constructed questionnaire); only applicable for parents/legal guardian of the immediate treatment group

Parents/legal guardians fill out the general questionnaire again at T1 to administer changes regarding current treatment.

Children:

- Planning (using a neuropsychological task; tower test)
- Social skills (using SCVT)
- Working memory (using a neuropsychological task; chessboard task)
- Self-efficacy (using a self-constructed questionnaire)

- Satisfaction regarding HealSeeker (using a self-constructed questionnaire); only applicable for children of the immediate treatment group

Teacher:

- Time management (using a self-constructed questionnaire)
- Planning (using the planning and organising subscale of the BRIEF)
- Social skills (using a subscale of the SSIS-RS)
- Working memory (using the working memory subscale of the BRIEF)

The questionnaire concerning satisfaction of the intervention will be given to children and their parents/legal guardians to assess the degree of satisfaction on HealSeeker. For the immediate treatment group this will be at T1. For the delayed treatment group this will be at T2. The assessor should preferably be blinded for the distribution of this questionnaire. In practice this means someone else of the trial team other than the assessor should take this satisfaction questionnaire, if possible. In this way the assessor will not be influenced by knowing if the child played or did not play the game. The assessor will be asked if he/she has the idea the child played or not, to investigate whether this blinding is broken or not. As the trial is open, it is not mandatory to keep the assessor blinded and information about whether assessor was blinded will be collected.

T2 Measurements/T2 visit

To assess the level of skills 10 weeks after the end of the intervention, the following measurements will be done:

Parent/legal guardian:

- Time management (using a self-constructed questionnaire and the It's about time questionnaire)
- Planning (using the planning and organising subscale of the BRIEF)
- Social skills (using a subscale of the social skills rating scale)
- Working memory (using the working memory subscale of the BRIEF)

Parents/legal guardians fill out the general questionnaire again at T2 to administer changes regarding current treatment.

Children:

- Planning (using a neuropsychological task)
- Social skills (using a social cognitive test)
- Working memory (using a neuropsychological task)
- Self-efficacy (using a self-constructed questionnaire)

Teacher:

- Time management (using a self-constructed questionnaire)
- Planning (using the planning and organising subscale of the BRIEF)
- Social skills (using a subscale of the social skills rating scale)
- Working memory (using the working memory subscale of the BRIEF)

Two reminders are sent by the eCRFsystem if these questionnaires for teachers are not filled out within a predefined time frame after T0, T1 and T2. Parents/legal guardians will give permission to the investigators to contact the child's teacher through the written informed consent. On the general questionnaire, parents/legal guardians can fill out the contact information of the teacher, so he/she can be asked to also fill out some questionnaires about the child. The teachers will receive an informational letter explaining the study and the importance of their experiences with the child in the classroom. The teacher will be asked to fill in several questionnaires at the measurement moments T0, T1 and T2. The teacher's questionnaires are on a protected internet site, which teachers can reach through a link in an e-mail they will receive.

3.5 Burden of participants

Parents/legal guardians and the child will need to fill in some questionnaires before and after the intervention. In addition, the child will take some neuropsychological tests. The approximate time investment per measurement moment for parents/legal guardians is given in Table 2. Approximate time investment for the child is given in Table 3. Time spent playing HealSeeker is not mentioned in the table. Each child is encouraged to play 3 times a week for a maximum of 45 minutes per game moment. Approximate time investment for the teachers is given in Table 4. Questionnaires concerning the diagnosis for ADHD and co morbidity, severity, game experience and satisfaction will only be taken once. The two subtests of the WISC-III (Wechsler, 2005) is optional and therefore not depicted in the table.

Table 2. *Time investment of parents/legal guardian for this study*

Questionnaires per testing moment	Time investment (min)	
Diagnostic interview (K-SADS)	90-120 min	
Vragenlijst voor gedragsproblemen bij Kinderen (VvGK)	20 min	
General questionnaire	5 min.	x1
Time management questionnaire	5 min.	x3
“It’s about time” questionnaire	10 min.	x3
BRIEF parent (subscale planning and organising and working memory)	10 min.	x3
Social Skills Improvement System (SSIS) Rating Scales (subscale social skills)	15 min.	x3
Game experience questionnaire	5 min.	
Satisfaction questionnaire	5 min.	
**Total:**	245 - 275 min.	

Table 3. *Time investment of the child for this study.*

Questionnaires per testing moment	Time investment (min)	
Self-efficacy scale	5 min.	x3
Social Cognitive Skills Test (SCVT)	30 min	x3
Tower Test	20 min	x3
Chessboard Task	15 min	x3
Satisfaction questionnaire	5 min	
**Total:**	215 min.	

Table 4. *Time investment of the teacher for this study*

Questionnaires per testing moment	Time investment (min)	
Time management questionnaire	5 min.	x3
BRIEF parent (subscale planning and organising and working memory)	10 min.	x3
Social Skills Improvement System (SSIS) Rating Scales (subscale social skills)	15 min.	x3
**Total:**	90 min.	

For the child, each measurement moment will not take more than 75 mintues with a 15 minute break. Regardless of the condition the child is in, each playing session will last a maximum of 45 minutes. Children will play three times a week, which adds up to 45 x 3 = 135 minutes per week of playing (during intervention this is 135 x 10 = 1080 minutes). Apart from playing the game, the children in the intervention condtion can play in the social community, which is about sixty minutes a week (3 x 20 minutes) or 480 minutes in 10 weeks. It is expected that children will regard playing the game as a small burden, because children with ADHD tend to enjoy playing such computer games. Also, no traveling time is needed to play HealSeeker, since it can be played at home. The children are therefore free to choose, in accordance with their parents/legal guardians, when they want to play.

4. STUDY POPULATION

4.1 Sample

This study will include a total of 170 children with ADHD, randomly selected among the research sites in The Netherlands and Belgium.

4.2 Inclusion criteria

Potential subjects (children and their parents) must satisfy all of the following criteria to be enrolled in the study:

1. All children included in the study will be from 8 to 12 years of age.
2. All children must have an official DSM-IV ADHD diagnose (all subtypes). The diagnosis will be assessed using the Kiddie-SADS Present and Lifetime version. Children with common diagnosed comorbid disorders (i.e. dyslexia, ODD) can participate in the study. Including children with comorbid disorders is both pragmatic because these comorbidities are very common among children with ADHD, and clinically relevant because the included children will be representative of the clinical population.
3. All children need to be stable on ADHD treatment, both pharmacological and psychological, for at least two months prior to start of the intervention. Stable treatment can also include no pharmacological and psychological treatment. Participants must, when possible, continue this treatment throughout the intervention period and between measurement moments. All types of treatments, both pharmacological and psychological are accepted. Whether a child is on stable treatment will be assessed by interviewing parents, who have the best overview of children’s treatment and actual use of treatment.
4. Minimum total intelligence score must be 80. If the total intelligence score is not known, has been established by a non-COTAN approved test or has been performed more than two years previous to the start of the intervention, total intelligence score will be established using two subtests of the Wechsler Intelligence Scale for Children third version (WISC-III-NL; Wechsler, 2005).
5. Children can only be included after a written informed consent has been signed by both parents or legal guardians. It is important that parents understand the information and are able to fill out the consent form. Twelve year olds have to give their own written informed consent in addition to their parents/legal guardians.
6. Both children and at least one of the parents/legal guardians must have a reasonable understanding of the Dutch language in order to understand the messages in the game, and have clear communication with the researchers.

4.3 Exclusion criteria

Potential subjects (children) who meet any of the following criteria will be excluded from participating in the study. These criteria will be assessed by interviewing the parents. If in doubt for exclusion criteria 2, the K-SADS interview modules are applied.

1. Children with a severe physical (i.e. Developmental Coordination Disorder), auditory (i.e. deafness), visual (i.e. blindness), neurological (i.e. epilepsy), speech and language (i.e. expressive receptive language disorder) or cognitive (i.e. mental handicap) disability will encounter great difficulties in playing HealSeeker, as will children with severe dyslexia (if they are not able to read texts), and are problematic for standardised measurements. These children will be excluded from participation.
2. Furthermore, children who are addicted to drugs, alcohol and/or gaming, have conduct disorder (CD) or have severe acute psychiatric disorders, psychotic disorder, major depressive disorder and mania will be excluded.
3. Children with an Autism Spectrum Disorder and Pervasive Developmental Disorder – Not Otherwise Specified will be excluded.
4. Children who have previously played HealSeeker (e.g. in the pilot study) are excluded.

4.4 Other conditions

Children must have access to a computer with internet and sound. The computer has to date from end 2006 / begin 2007. The following minimal system requirements are formulated: (1) Windows XP Service Pack 3 / Windows Vista / Windows 7, (2) Dual Core 2.0 GHz or equivalent processor, (3) 1 GB RAM (Windows XP) or 2 GB RAM (Windows Vista / Windows 7), (4) Flash Player 11.3, (5) latest version of Internet Explorer, Firefox or Chrome browser (6) and a minimum screen resolution of 1280 x 960.

4.5 Sample size calculation

In the HealSeeker pilot study the Total score on the time management scale (range 11 to 110) rated by the child's parent showed a mean (SD) improvement versus Baseline of +8.6 (13.3) at Week 8 in the intervention group (high frequent play condition) versus -1.3 (19.9) in the control group (low frequent play condition). Assuming a SD of 16 for change from Baseline in Total score a sample size of 78 per group would allow detection of a difference of 8 points at 5% two-sided significance level with 87% power. To account for dropouts 85 subjects per group will be enrolled. The study will also be sufficiently powered for the other primary outcome measures assuming effect sizes of at least 0.5.

Hochberg procedure (Biometrika,1988) will be applied to preserve the family-wise type I error rate (alpha) at 5%.

5. TREATMENT OF SUBJECTS

5.1 Description of intervention

HealSeeker game

HealSeeker is an online computer game with a futuristic and adventurous character. Several missions and three minigames are embedded in the game. These minigames are related to three learning goals: time management, planning and organisation and social behaviour.

Story

When playing HealSeeker the player takes on the role of a space captain. It's a dangerous but exciting job! Throughout the game the player is assigned to various different missions by his commander Alzor Hubble. Alzor has worked for the BOEM agency for many years and is held in high esteem. He will guide the player, give him/her feedback and help wherever he can. BOEM's goal is collecting and recovering rare minerals. These lay scattered throughout the universe and are often found at dangerous sites such as caves or sewers.

Missions

HealSeeker is divided into a number of different missions and side-missions (see Table 5). Each mission has different tasks the player has to complete. Once a mission is completed by the player, the next missions will become available.

Table 5. *Description of missions embedded in HealSeeker.*

**Mission number**	**Mission name**
Mission 00	Go and see the commander!
Mission 01	Find a scientist for your team
Mission 02	Find a cultural expert for your team
Mission 03	Find a mechanic for your team
Mission 04	Get your space travel certificate
Mission 05	Decipher the ancient tablet
Mission 06	Inquire about the old legends
Mission 07	Find the sacrifice for the second wise man!
Mission 08	Find the sacrifice for the third wise man!
Mission 09	Explore the origin cave

Minigames

A minigame is a small game within the larger game environment. Every minigame is isolated and stands on its own, which makes it possible to offer the player unique game elements. This makes minigames specifically useful for integrating learning goals. HealSeeker includes three minigames that addresses several learning goals that are important for children with ADHD (see Table 6). These learning goals are implicitly interwoven in the game.

Table 6. *HealSeeker minigames and learning goals*

**Minigames**	**Learning goal**
Labyrinth	Time management
Explorobot	Planning and organizing
Space Travel Trainer	Social functioning

These above mentioned learning goals will supplementary be trained within the larger game environment during missions and side-missions. As such, minigames and the larger game environment are complementing each other. Further general learning goals within HealSeeker are listening to the mentor, deal with frustration, ignore distraction, learn to concentrate, be attentive and inhibit impulses.

Progression

Every minigames starts with a tutorial level in which game principles are explained to the player. Players can make progress in the game by succeeding levels within the minigames. Levels and missions become more difficult as the player progresses. The player will visit several planets during his missions.

Social community

HealSeeker includes a closed social community in which children can communicate with each other through predefined messages. Children can look into each others profile and see each others space ship. To stimulate social behaviour, children are able to ask each other for help. Finally, it will be possible to see how much progression children make during the game.

Space ship editor

Once the player is able to access his/her space ship he/she will find the space ship editor. This is an application in the game in which the player is able to customize the ship to his/her liking. Through the game the player will find several items for his spaceship, but he can also buy items in shops on the different planets.

Shops

The player will find shops in the game. In those shops he/she is able to purchase items and sell them. Shops can be found on different planets. The interface shows which items the player can afford and will give descriptions so the player knows more about the item. In different shops the player will find different items.

Inventory

In the game the player find items. These items are stored in his inventory. The player can use items, described below, in the shops to sell them or in the space ship editor. The inventory items can be categorized in the following four groups: minerals, ICO upgrades, rocket parts and items.

Player profile

The player will have a player profile within the game. In this profile a picture of the players avatar can be found, together with a list of all the missions the player has completed. Also a view of the players current spaceship is visible.

Non-player characters (NPCs)

HealSeeker includes several NPCs that are not controlled by the player.

- Regulus Vlampijp: Regulus Vlampijp is the bus driver which will bring the player to earth. At the beginning of the game he is the players mentor.
- Alzor Hubble: Alzor Hubble is a commander at BOEM headquarters. Through the game Alzor will be the players mentor. The player can contact him for any questions or inquiries.
- ICO: ICO is the robot which is designed especially for space captains to assist them in their missions. ICO's head is a big monitor on which the player can contact his members.
- Brutus Protonus: Brutus Protonus is a Cave instructor. He will explain the mechanics of the Labyrinth game to the player and will guide the player through the levels.
- Kortar Nebuloid: Kortar is the scientist for the players team. He studied minerals all his live and is able to provide the player with useful information if needed.
- Violet Velorum: Violet is the head of the science department on earth. If the player wants to trade minerals for Quarks (i.e. the payment unit) she is the person to go to.

- <u>Nika Syntax:</u> Nika is the cultural expert for the players team. She has studied a lot with books, but lacks the practical experience. She knows 36 languages and will provide the player with useful information about and where needed.
- <u>Vesto Tesla:</u> Vesto is the mechanic for the players team. He can fix anything and will use his skill to the players benefit. Vesto likes to experiment, which doesn't always work out.
- <u>Pluton Verne:</u> Pluton is the Space Travel instructor. He will teach the player to fly a spaceship. If the player does well enough he will give him his Space Travel Certificate.
- <u>Nikolar Nebuloid:</u> Nikolar is a wise old professor and a grandfather to Kortar. Nikolar helps the player decipher the mysterious tablet.
- <u>Pandemia Quaksala:</u> Pandemia is the nurse in charge of Nikolars health. She asks the player to find the rare medicine to cure Nikolar from his disease.
- <u>Guru 1:</u> Guru is a spiritual person which the player encounters on the planet matata-12. He tells the player he lives for the spiritual realm, but is in need of some material items.
- <u>Guru 2:</u> Guru is a spiritual person which the player encounters on the planet Krikkit. He is specialized in making tea.

Involvement of investigators

Because HealSeeker is an online game, this game is offered at the child's home and therefore played independently from the investigators during the research period. Every child has her/his own login name and password. During the measurements moments children and their parents/legal guardians have to fill out several questionnaires. During these measurements they are supervised by a investigator with a master degree in pedagogy or psychology. Online questionnaires will be offered at a laptop in a testing room that is quite and free of distractions.

Involvement of parents/legal guardians

Information meetings can be organised before and after the intervention period. Parents/legal guardians from the participants will receive information regarding the content of the study and are introduced to management strategies for their children's game behaviour. It is important to inform parents/legal guardians, making sure that there will be no worries about the study and prevent that they will interfere with the intervention. During these information meetings it will be emphasised again that participation is voluntary and that the child can withdraw himself/herself anytime. Withdrawal from the study will not influence current

treatment children receive. All parents/legal guardians receive memory game cards (with pictures of the game) before the start of the intervention. They are instructed to give their child one of these cards after each game moment. At the end of the intervention period an information meeting can be organised to evaluate and share parents'/legal guardians' experiences, depending on the interest for such a meeting. Because HealSeeker is a new approach to train skills in children with ADHD, it is expected that there is a pressing need for these information meetings.

5.2 Use of co-intervention

In addition to treatment as usual (i.e. pharmaceutical, cognitive-behavioural therapy and/or psycho educational sessions), all children will receive the HealSeeker game during the research period. The first group will immediately start playing Healseeker after baseline measurements and the second group will start 10 weeks later. The treatment as usual is offered by the mental health care institution where the child is registered. This treatment has to be stable for two months while the study continues and preferably between measurement moments. The treatment as usual will be registered and monitored by a general questionnaire which is answered by the parent/legal guardian.

6. METHODS

6.1 Study parameters/endpoints

6.1.1 Screening measures

Screening measure: Intelligence

Instrument: Wechsler Intelligence Scale for Children III (WISC-III)

Children must meet the following criterion: TIQ ≥ 80. A previous assessment of IQ dating back less than 2 years with tests being comparable with the WISC-III must be available. If no assessment of IQ dating back less than 2 years is available, this criterion will be checked by administering two subtests (i.e. vocabulary and block design) of the Wechsler Intelligence Scale for Children III (WISC-III) (Wechsler, 2005). The norm score of the subtest will be multiplied with 5 and summed up. Afterwards with this total score the TIQ can be estimated. This composite score has satisfactory reliability (*r* = 0.91) and correlates highly with the full-scale IQ score (Dovis et al., 2010; Sattler, 2001).

Screening measure: DSM-IV ADHD diagnosis and co morbidity

Instrument: Kiddie-SADS-Present and lifetime version (K-SADS)

The K-SADS (Puig-Antich & Chamber, 1978; Reichart, Wals & Hillegers, 2000) is a semi structured interview designed to diagnose psychiatric problems using the DSM-IV criteria. For this study the parents are interviewed to reconfirm the ADHD diagnosis and guarantee the ADHD diagnosis is made in a standardized way. In addition, children who are addicted to drugs, alcohol and/or gaming, have CD or severe acute psychiatric disorders such as psychotic disorder, major depressive disorder or mania will be excluded on basis of an interview with parents and in doubt this will be checked with the K-SADS. Duration of the interview is about 30 minutes.

Screening measure: Severity of symptoms

Instrument: VvGK filled out by parents/legal guardians **(Attachment A.)**

The VvGK is originally developed by Pelham, Gnagy, Greenslade and Milich (1992) and translated to Dutch by Oosterlaan and colleagues (2000). It is a questionnaire which contains both a parent and a teacher version. For this study only the parent version will be used. The VvGK measures if and to what extent symptoms of ADHD, ODD and CD are present in children and can be used as a diagnostic tool. It consists of 42 items measuring in four domains: attention deficit, hyperactivity and impulsivity, oppositional behaviour, and conduct disorders. Completing the questionnaire takes about ten minutes. Good psychometric values

are reported by the COTAN: Norms: sufficient, Reliablity: sufficient, Construct validity: good, Criterium validity: not researched.

Screening measure: General child characteristics
Instrument: Self-constructed questionnaire filled out by parents/legal guardians **(Attachment.B)**
To collect information about the background and medical history of the children a general questionnaire is given to the parents/legal guardians. There is also a question in this questionnaire about the current support for the parents/legal guardians (such as psycho education or parent support groups).

6.1.2 Outcome measures

Outcome measure: Planning
Instrument: BRIEF filled out by parents/legal guardians and teachers (subscale: Plan/Organise) **(Attachment.C – parents and Attachment I - teachers)**
The Behaviour Rating Inventory of Executive Function (BRIEF) is a screening list to assess executive functioning in the home and school environment in children 5-18 years of age (Smidts & Huizinga, 2009). The questionnaire contains 86 items in eight non-overlapping clinical scales and two validity scales. These theoretically and statistically derived scales form two broader Indexes: Behavioural Regulation (three scales: Inhibit, Shift, Emotional Control) and Metacognition (five scales: Initiate, Working Memory, Plan/Organise, Organisation of Materials, Monitor), as well as a Global Executive Composite score. The answers are scored on a 3-point Likert scale (never – sometimes - often). It takes approximately 30 minutes to fill in the questionnaire. The Dutch version of the BRIEF contains norms of a representative sample of children in regular education. The BRIEF has a good reliability and construct validity (Elling & Minderaa, 2010).

Outcome measure: Planning
Instrument: Tower Test performed by the child
The Tower Test is a subtest of the Delis-Kaplan Executive Function System battery and tries to measure several executive functions such as spatial planning, rule learning, inhibition of impulsive responding and establishing and maintaining instructional set in children and adults from 8 to 89 years. The material consist of 5 disks that vary in size from small to large and a board with three vertical pegs. The objective is to move all the disks to the rod on the right, observing the following rules: only one disk may be moved at a time, a larger disk may not be placed on top of a smaller disk and disks must always be placed on one of the rods. Children

have to build a tower in as few as possible steps and as quickly as possible. Outcomes vary from number of rule violations, total number of moves to completion time, whereby various ratio's can be calculated. It takes around 30 minutes to administer the test. This test is not judged by the COTAN but research into the Tower of London Test indicates that this is a reliable and valid method to measure planning skills in children (Unterrainer et al., 2004).

Outcome measure: Time management
Instrument: Self constructed questionnaire filled out by parents/legal guardians and teachers **(Attachment.D – parents and Attachment I - Teachers)**
The time management scale contains 11 descriptions of time management behaviour. Parents/legal guardians and teachers are asked to rate the time management skills of their child on a 10 point Likert scale (ranging from true – not true). From the pilot study results it appeared that this questionnaire is reliable (Chronbach's alpha = 0.85).

Instrument: "It is about time" questionnaire (Barkley, 1998) filled out by parents/legal guardians **(Attachment.E)**
The "It is about time" questionnaire consist of 25 questions concerning time perception and time management. Parents/legal guardians and teachers are asked to rate the time perception and time management skills of their child. Completing the questionnaire takes about ten minutes.

Outcome measure: Social behaviour
Instrument: Social Skills Improvement System (SSIS) Rating Scales (subscale social skills) filled out by parents/legal guardians and teachers **(Attachment.F – parents and Attachment I - Teachers)**
The Social Skills Improvement System (SSIS) Rating Scales assists (Gresham & Elliot, 2008) in screening and classifying children with significant social skills deficits. There are 3 different rater forms: parent, teacher and student. The SSIS rating Scales assesses 3 domains: 1) Social Skills, 2) Problem Behaviour and 3) Academic Competence. The Social Skills questions are divided in 7 subdomains: communication, cooperation, assertion, responsibility, empathy, engagement self-control. The Problem Behaviour questions are divided over 5 subdomains: externalizing, bullying, hyperactivity/inattention, internalizing, autism spectrum. The SSIS rating scales appeared to have a high internal consistency reliability, as well as a high test retest reliability.

Outcome measure: Social behaviour
Instrument: Social-Cognitive Skills Test (SCVT) performed by the child.

This instrument measures eight social cognitive skills of children between 4 and 12 years of age. The eight age-related social cognitive skills are: Identifying, Discriminating, Differentiating, Comparing, Perspective-taking, Relating, Coordinating and Taking into account (Van Manen, Prins & Emmelkamp, 2001). This test consists of 7 short stories with corresponding pictures and each story contains 8 questions that represent the eight social cognitive skills. It takes approximately 30 minutes to complete the test. For this study it is decided to use the short version of the SCVT (version A), consisting of three stories. The reliability and construct validity of SCVT are judged as sufficient by the COTAN who asses the psychometric properties of the Dutch versions of psychological tests.

Outcome measure: Working memory

Instrument: Chessboard task performed by children

The chessboard task is a recently developed working memory performance task on the computer based on the Letter – Number sequencing task from the Wechsler Adult Intelligence Scale (WAIS; Wechsler, 1958) and the Corsi Block Tapping Task (Corsi, 1972). The task assesses the ability to manipulate/reorganise and maintain visual-spatial information relevant for the task at hand (Dovis et al, 2011). On the computer screen, a sequence of stimuli (squares that light up) is presented one by one on a four by four grid with green and blue squares in a chessboard formation. Each stimulus lights up for 900ms, after which a 500ms inter-stimulus interval is presented. The participants responds by clicking on the lit up squares, ordering the presented stimuli by clicking on the green squares first, and then the blue squares. The time it takes to administer this test depends on the amount of trials presented. Psychometric characteristics are unknown.

Outcome measure: Working memory

Instrument: BRIEF filled out by parents/legal guardians and teachers (subscale: Working memory) **(Attachment.G)**

The Behaviour Rating Inventory of Executive Function (BRIEF) is a screening list to assess executive functioning in the home and school environment in children 5-18 years of age. The questionnaire contains 86 items in eight non-overlapping clinical scales and two validity scales. These theoretically and statistically derived scales form two broader Indexes: Behavioural Regulation (three scales: Inhibit, Shift, Emotional Control) and Metacognition (five scales: Initiate, Working Memory, Plan/Organise, Organisation of Materials, Monitor), as well as a Global Executive Composite score. The answers are scored on a 3-point Likert scale (never – sometimes - often). It takes approximately 30 minutes to fill in the questionnaire. The Dutch version of the BRIEF contains norms of a representative sample of

children in regular education. The BRIEF has a good reliability and construct validity (Elling & Minderaa, 2010).

Outcome measure: Self-efficacy

Instrument: Self-constructed questionnaire filled out by children **(Attachment.H)**

This questionnaire is designed to get a better understanding of the kind of things that are difficult for children with ADHD. Children are asked to rate on a scale from 0 to 100 how certain they are that he or she masters certain skills concerning time management, planning and social behaviour.

6.1.3 Primary outcome measures

The three primary outcome measures are:

- Planning / organisation – measured by BRIEF (subscale plan/organise), total score of the parents;
- Time management – measured by self-constructed questionnaire, total score of the parents;
- Social behaviour – measured by SSIS (subscale cooperation), total score of parents.

6.1.4 Additional measures

Exploratory in-game measurements

HealSeeker is attached to a database which files the anonymised game results of the children. Examples of variables that are measured are play date, play time and within game progression.

Baseline measurement of game experience and use

Instrument: Self-constructed questionnaire filled out by parents/legal guardians **(Attachment.J)**

This questionnaire examines the experience level of children concerning gaming and computer use.

Post-test measurement of satisfaction

Instrument: self-constructed questionnaire filled out by parents/legal guardians and children **(Attachment.K – parents and Attachment L - Children)**

Parents and children can indicate how satisfied they are about the

game on the satisfaction questionnaire. Answers are scored on a 10-point Likert scale.

6.2 Randomisation, blinding and treatment allocation

Randomisation will be used to minimize bias in the assignment of subjects to treatment groups to increase the likelihood that known and unknown subject characteristics (e.g., demographic and baseline characteristics) are equally balanced across treatment groups (1:1), and to enhance the validity of statistical comparisons across treatment groups.
The randomization list will be stratified by site and gender using randomly permuted blocks. Randomisation to the immediate and delayed treatment group condition will occur after collection of baseline data and confirmation of eligibility in the web-based ECD system.
The randomization process will be automated and based on a pre-specified computer-generated randomization list. A randomization number will be assigned online using the next available number on the randomization list corresponding with the site and gender of the subject. The trial will be open-label. Due to the nature of the intervention and time frame, it will be obvious for participants, their parents and teachers and the site staff to which treatment group they are allocated. All efforts are made to keep the assessor blind. If a child spontaneously tells the assessor his or her treatment condition, this will be registered.

6.3 Signaling resistance

Measurements

Possible (nonverbal) resistance of children during measurements will be evaluated in different manners. The investigator will observe children on signals of restlessness and/or emotional reactions as a consequence of filling out the questionnaires. Examples of emotional reactions are anger, sadness, fear or panic. In case of such emotional reactions the investigator will try to find out where this resistance is coming from and will calm down the child for example by giving more information about the trial. In addition it will be explained to the child that he/she does not have to fill out the questionnaires if he/she does not want to do that. Measurements will be registered as discontinued as a consequence of resistance (see also “Behaviour code of resistance with regard to participation in medical scientific research” at www.ccmo.nl established by the Dutch National Association of Paediatricians). The investigator will emphasize that the child can leave the study at any time for any reason if they wish to do so without any consequences. During these measurements children are supervised by a investigator who has a master degree in pedagogy or psychology. They are able to recognise restlessness and/or emotional reactions and will be alert on these signals during measurements.

Intervention period

Parents/legal guardians will be informed about recognising signals of resistance and how to cope and react on it. In addition, parents/legal guardians will be individually informed by the investigator during the T0 measurement about how they think their child will react on the game and what form of resistance they expect. Parents/legal guardians and investigator decide upon the way parents/legal guardians will cope and react on the resistance of their children. It is possible that the investigator performs a home visit in which he/she explains the child how the game works. As such the child may be less fearful to play the game at home. When a child does not want to play the game and shows signals of restlessness or resistance, study participation will be registered as discontinued as a consequence of resistance. During the intervention period investigators are available through mail and by phone to answer questions parents/legal guardians may have. It is expected that resistance is minimal in this study during the intervention period because these children are highly motivated to play a serious game. Through good coaching and availability of the investigator resistance can be minimised. The investigator will emphasise that parents/legal guardians and their child can leave the study at any time for any reason if they wish to do so without any consequences.

6.4 Withdrawal of individual subjects

Parents/legal guardians and children can leave the study at any time for any reason if they wish to do so without any consequences. This is also explicitly formulated in the information letter for parents/legal guardians and children. It will be stressed that withdrawal from the study does not have any consequences for the treatment as usual children follow within their mental health care institution. In the case that parents/legal guardians and children decide to leave the study, this means that no further information will be gathered from the child. It is expected that withdrawal is minimal in this study during the intervention period because these children are highly motivated to play a serious game. Through good coaching and availability of the investigator withdrawal can be minimised. Withdrawal will be registered as an indication of treatment compliance.

6.5 Replacement of individual subjects after withdrawal

Subjects will not be replaced after withdrawal.

6.6 Follow-up of subjects withdrawn from treatment

No follow-up will be conducted with subjects withdrawn from treatment.

6.7 Premature termination of the study

Parents/legal guardians and children have to fill out several questionnaires. Children participate in the study for a period of 10 weeks. It is expected that these measurements and interventions do not involve any risks for the participants. Therefore no criteria are formulated for premature termination of the study.

7. SAFETY REPORTING

Timely, accurate, and complete reporting and analysis of safety information from clinical studies are crucial for the protection of subjects, investigators, and the sponsor, and are mandated by regulatory agencies worldwide. The sponsor has established Standard Operating Procedures in conformity with regulatory requirements worldwide to ensure appropriate reporting of safety information; all clinical studies conducted by the sponsor or its affiliates will be conducted in accordance with those procedures.

All adverse events will be reported from the time a signed and dated informed consent form is obtained until completion of the subject's last study-related procedure. An adverse event is any untoward medical occurrence in a clinical study subject administered a procedure. An adverse event does not necessarily have a causal relationship with the treatment. An adverse event can therefore be any unfavorable and unintended sign (including an abnormal finding), symptom or disease temporally associated with the use of a procedure, whether or not related to that procedure. However, for this study we will only collect adverse events that are possibly, probably or very likely related to the study procedures.

The Sponsor will evaluate any safety information that is spontaneously reported by an Investigator beyond the time frame specified in the protocol.

8. STATISTICAL ANALYSES

The modified ITT analysis set will include all randomized subjects who have both a baseline and at least one post-baseline efficacy assessment. Hochberg procedure (Biometrika, 1988) will be used to control the experiment-wise alpha level at 0.05 for the multiple primary endpoint analyses. All tests will be two-sided.

For all outcome measures descriptive statistics (mean, SD, median and range) as well as their changes from T0 (Baseline) will be calculated by treatment group. Within-group comparisons of changes at T1 (Week 10) from T0 will be conducted in both groups.

To assess whether effects are maintained after stopping the game, within-group comparisons of changes at T2 (Week 20) versus T1 will be performed in the Immediate group. To assess effects of game after waiting period, within-group comparison of changes at T2 versus T1 will be conducted in the delayed group.

An analysis of covariance (ANCOVA) model that includes treatment, site and gender as factors and baseline as covariate, will be used for the primary outcome measures to estimate the difference between the two groups in changes from T0 at T1.

Exploratory analyses may be conducted as deemed appropriate to examine whether intervention effects vary as function of demographic and baseline characteristics (e.g. age, gaming experience), to explore correlations between different measures, to investigate potential mediating effects (e.g. working memory, self-efficacy).

All adverse events will be listed. Special attention will be given to those subjects who discontinued due to an adverse event.

9. ETHICAL CONSIDERATIONS

9.1 Regulation statement

The current study does not concern a medical intervention but a study to on the effectiveness of the HealSeeker as a cognitive and behavioural intervention. The goal of this training is to enhance time management, organisational skills, planning and social skills in children with ADHD through a computer game with built-in learning strategies. Some principles of the declaration of Helsinki (Seoul, 2008) are applicable to this study. According to this declaration, the primary goal of medical research involving humans is improving preventive, diagnostic and therapeutic procedures and understanding the aetiology and pathogenesis of an illness/disorder. The parents/legal guardians and children will be correctly informed about the goals, methods and possible discomforts of the training. Also, all participants and their parents/legal guardians will be informed on their right of denying participation or withdrawal at any moment in the study, without any further consequences (World Medical Association Declaration of Seoul, 2008). The study will be conducted according to the principles of the Declaration of Helsinki (Seoul, 2008) and in accordance with the Medical Research Involving Human Subjects Act (WMO) for the Netherlands and Wet Inzake Experimenten op de Menselijke Persoon for Belgium and other guidelines, regulations and Acts.

9.2 Recruitment and consent

Participants will be children with ADHD 8 to 12 years old, recruited through the research site in the Netherlands and Belgium. The investigator will approach the parents/legal guardians and the children and shortly inform them about the research. When parents/legal guardians and the children are interested in participating in the study, they are invited for a screening visit/ T0 visit (prior to randomization), where the study is further explained. If parents/children want they can also receive an envelope containing the informed consent for the parents/legal guardians, an informational pamphlet (informed assent) for the child and a written informed consent at home. The information is about how study to the learning effects of a Serious Game will be conducted. In addition, parents/legal guardians and their child will be invited to participate in the study. Both parents/legal guardians and twelve-year-olds will be asked to sign the written informed consent. The informed consent/ assent clearly states that participants are allowed to withdraw at any moment in the study without further consequences or reason. The practitioner will emphasise that choosing to join or not will not affect in any way the treatment the child is currently receiving at the mental health care institution. In accordance with the Dutch WMO and Belgium law (Wet Inzake Experimenten op de Menselijke Persoon), parents/legal guardians will have to sign a written informed consent. Parental consent is crucial for joining the study. Parents/legal guardians have to

give their consent for the approach of the teacher by the researchers. If the participant is twelve years of age he or she will have to sign an informed consent, in addition to their parent. The informed consent obtained from the parent or his or her legally acceptable representative includes explicit consent for the processing of personal data and for the investigator/institution to allow direct access to his or her original medical records (source data/documents) for study-related monitoring, audit, IEC/IRB review, and regulatory inspection. This consent also addresses the transfer of the data to other entities and to other countries. The researcher will sign all informed consents. After receiving the signed informed consent, parents/legal guardians will be contacted by phone to decide if an IQ test is needed, and appointments will be made for the measurement moments.

9.3 Investigator Responsibilities

The investigator is responsible for ensuring that the study is performed in accordance with the protocol, current ICH guidelines on Good Clinical Practice (GCP), and applicable regulatory and country-specific requirements. Good Clinical Practice is an international ethical and scientific quality standard for designing, conducting, recording, and reporting studies that involve the participation of human subjects. Compliance with this standard provides public assurance that the rights, safety, and well-being of study subjects are protected, consistent with the principles that originated in the Declaration of Helsinki, and that the study data are credible.

9.4 Independent Ethics Committee or Institutional Review Board

Before the start of the study, the sponsor will provide the IEC/IRB with current and complete copies of the following documents (as required by local regulations):

- Final protocol and, if applicable, amendments
- Sponsor-approved ICF/Assent (and any other written materials to be provided to the subjects)
- Sponsor-approved parent recruiting materials
- Information on compensation for study-related injuries or payment to subjects for participation in the study, if applicable
- Investigator's curriculum vitae or equivalent information (unless not required, as documented by the IEC/IRB)
- Information regarding funding, name of the sponsor, institutional affiliations, other potential conflicts of interest, and incentives for subjects
- Any other documents that the IEC/IRB requests to fulfill its obligation

This study will be undertaken only after the IEC/IRB has given full approval of the final protocol, amendments (if any, excluding the ones that are purely administrative, with no

consequences for subjects, data or study conduct), the ICF, applicable recruiting materials, and parent compensation programs, and the sponsor has received a copy of this approval. This approval letter must be dated and must clearly identify the IEC/IRB and the documents being approved. During the study the investigator (or sponsor where required) will send the following documents and updates to the IEC/IRB for their review and approval, where appropriate:

- Protocol amendments (excluding the ones that are purely administrative, with no consequences for subjects, data or study conduct)
- Revision(s) to ICF and any other written materials to be provided to subjects
- If applicable, new or revised parent recruiting materials approved by the sponsor
- Revisions to compensation for study-related injuries or payment to subjects for participation in the study, if applicable
- Summaries of the status of the study at intervals stipulated in guidelines of the IEC/IRB (at least annually)
- Any other requirements of the IEC/IRB

For all protocol amendments (excluding the ones that are purely administrative, with no consequences for subjects, data or study conduct), the amendment and applicable ICF revisions must be submitted promptly to the IEC/IRB for review and approval before implementation of the change(s). At least once a year, the IEC/IRB will be asked to review and reapprove this study. The reapproval should be documented in writing (excluding the ones that are purely administrative, with no consequences for subjects, data, or study conduct). At the end of the study, the investigator (or sponsor where required) will notify the IEC/IRB about the study completion.

9.5 Objection by minors or incapacitated subjects (if applicable)

The study is aimed at children of 8 to 12 years of age. Parents/legal guardians have to sign a written consent, but only if their child is willing to participate. If the participant is twelve years of age he or she will have to sign an informed consent (assent), in addition to their parent. Signed informed consent from a parent/legal guardian is crucial for participation. Participants are free to withdraw from the intervention at any time, without further consequences. All participants will have a minimum IQ of 80.

9.6 Benefits and risks assessment, group relatedness

Both children and their parents/legal guardians will be well informed about the goals and used methods in this study, and of the advantages and disadvantages of joining, and brochures for parent and child have been made solely for this purpose. The risks of this

intervention are limited, especially since the study does in no way interfere with regular treatment or education of the children with ADHD. Thereby, the risk of game addiction is negligible. Game addiction occurs in situations of children playing games for more than 40 hours per week (Van Rooij, Schoenmakers, Meerkerk & Van de Mheen, 2009). The maximum game play time per day is set at 45 minutes for both HealSeeker, combined with 20 minutes in the social community (HealSeeker). Because of the limited available game time, the risk of addiction is minimal. In addition, most addictive games have a component of random rewards. This is not the case in HealSeeker in which only positive behaviour is rewarded. Finally, the prevalence rates of game addiction in children 13 till 16 years of age are very low and even lower in younger children. The risk of addiction is not very likely for the age group included in this study. The current study concerns a therapeutic intervention through a computer game. The goal of this training is to enhance time management, organisational skills, planning and social skills in children with ADHD through a computer game with built-in learning goals. The computer game can be played online and at home. The risks of participation can be considered negligible and the burden can be considered minimal. This study provides children with ADHD with the opportunity to join a non-medicinal intervention aimed at abating associated problems and elevating empowerment. Parents/legal guardians and the child can withdraw from the intervention at any moment, without questions or further consequences.

9.7 Compensation for injury

Based on the above mentioned risk taxation, for this study, we wish to obtain dispensation from the statutory obligation to provide insurance, because we presume participating in the study is without risk for which the insurance applies (attachment G1).

10. ADMINISTRATIVE ASPECTS AND PUBLICATION

10.1 Handling and storage of data and documents

All data gathered for this study will be handled anonymously and confidentially. Subjects will receive a code, based on the group they are in (intervention or control) and serial number. Gaming information will be gathered in an online database linked to the personal identification code of the participant. If necessary, data can be traced back to a participant using an identification code list. The main investigator is the only person with access to this list, and safeguards the anonymity of the list. Parents/legal guardians of the participants will be asked permission to keep the information for future research (attachment E2). This treatment of the obtained data conforms to the Law for Protection of Personal Data in the Netherlands and Belgium. In case of publishing, this data will be reported in group form and can in no way be traced back to the individual participants. Electronic Data Capture (eDC) will be used for this study. The electronic file will be considered to be the CRF. Worksheets may be used for the capture of some data to facilitate completion of the CRF. Any such worksheets will become part of the subject's source documentation. All data relating to the study must be recorded in CRFs prepared by the sponsor.

10.2 Amendments

All substantial amendments will be notified to the METC and to the competent authority.

Non-substantial amendments will not be notified to the accredited METC and the competent authority, but will be recorded and filed by the sponsor. Neither the investigator nor the sponsor will modify this protocol without a formal amendment by the sponsor. All protocol amendments must be issued by the sponsor, and signed and dated by the investigator. Protocol amendments must not be implemented without prior IEC/IRB approval, or when the relevant competent authority has raised any grounds for non-acceptance, except when necessary to eliminate immediate hazards to the subjects, in which case the amendment must be promptly submitted to the IEC/IRB and relevant competent authority. Documentation of amendment approval by the investigator and IEC/IRB must be provided to the sponsor. When the change(s) involves only logistic or administrative aspects of the study, the IRB (and IEC where required) only needs to be notified. During the course of the study, in situations where a departure from the protocol is unavoidable, the investigator or other physician in attendance will contact the appropriate sponsor representative (see Contact Information page(s) provided separately). Except in emergency situations, this contact should be made before implementing any departure from the protocol. In all cases, contact with the sponsor must be made as soon as possible to discuss the situation and agree on an

appropriate course of action. The data recorded in the CRF and source documents will reflect any departure from the protocol, and the source documents will describe this departure and the circumstances requiring it.

10.3 Regulatory Approval/Notification

This protocol and any amendment(s) must be submitted to the appropriate regulatory authorities in each respective country, if applicable. A study may not be initiated until all local regulatory requirements are met.

10.4 Required Prestudy Documentation

The following documents must be provided to the sponsor before study start:

- Protocol and amendment(s), if any, signed and dated by the principal investigator
- A copy of the dated and signed (or sealed, where appropriate per local regulations), written IEC/IRB approval of the protocol, amendments, ICF, any recruiting materials, and if applicable, subject compensation programs. This approval must clearly identify the specific protocol by title and number and must be signed (or sealed, where appropriate per local regulations) by the chairman or authorized designee.
- Name and address of the IEC/IRB, including a current list of the IEC/IRB members and their function, with a statement that it is organized and operates according to GCP and the applicable laws and regulations. If accompanied by a letter of explanation, or equivalent, from the IEC/IRB, a general statement may be substituted for this list. If an investigator or a member of the study-site personnel is a member of the IEC/IRB, documentation must be obtained to state that this person did not participate in the deliberations or in the vote/opinion of the study.
- Regulatory authority approval or notification, if applicable
- Documentation of investigator qualifications (eg, curriculum vitae)
- Signed and dated clinical trial agreement, which includes the financial agreement

Any other documentation required by local regulations

- The following documents must be provided to the sponsor before enrollment of the first subject;
- Documentation of subinvestigator qualifications (eg, curriculum vitae)

10.5 Subject Identification, Enrollment, and Screening Logs

The investigator agrees to complete a subject identification and enrollment log to permit easy identification of each subject during and after the study. This document will be reviewed by

the sponsor study-site contact for completeness. The subject identification and enrolment log will be treated as confidential and will be filed by the investigator in the study file. To ensure subject confidentiality, no copy will be made. All reports and communications relating to the study will identify subjects by subject identification and age. The investigator must also complete a subject screening log, which reports on all subjects who were seen to determine eligibility for inclusion in the study.

10.6 Source Documentation

At a minimum, source documentation must be available for the following to confirm data collected in the CRF: subject identification, eligibility, and study identification; study discussion and date of signed informed consent; dates of visits; results of efficacy parameters as required by the protocol; and date of study completion and reason for early discontinuation of study drug or withdrawal from the study, if applicable. In addition, the author of an entry in the source documents should be identifiable. At a minimum, the type and level of detail of source data available for a subject should be consistent with that commonly recorded at the study site as a basis for standard medical care. Specific details required as source data for the study will be reviewed with the investigator before the study and will be described in the monitoring guidelines (or other equivalent document).
The following data will be recorded directly into the CRF and will be considered source data:

- Questionnaires for parents/ legal guardians;
- Questionnaires for teachers;
- Results on tasks for children (may be added directly in the CRF, but also paper source can be used).

Inclusion and exclusion criteria not requiring documented medical history must be verified at a minimum by subject /parent interview.

10.7 Data Quality Assurance/Quality Control

Steps to be taken to ensure the accuracy and reliability of data include the selection of qualified investigators and appropriate study sites, review of protocol procedures with the investigator and study-site personnel before the study, and periodic monitoring visits by the sponsor. The sponsor will review CRFs for accuracy and completeness during on-site monitoring visits and after transmission to the sponsor; any discrepancies will be resolved with the investigator or designee, as appropriate. After upload of the data into the study database they will be verified for accuracy and consistency with the data sources.

10.8 Record Retention

In compliance with the ICH/GCP guidelines, the investigator/institution will maintain all CRFs and all source documents that support the data collected from each subject, as well as all study documents as specified in ICH/GCP Section 8, Essential Documents for the Conduct of a Clinical Trial, and all study documents as specified by the applicable regulatory requirement(s). The investigator/institution will take measures to prevent accidental or premature destruction of these documents. Essential documents must be retained until at least 2 years after the last approval of a marketing application in an ICH region and until there are no pending or contemplated marketing applications in an ICH region or until at least 2 years have elapsed since the formal discontinuation of clinical development of the investigational product. These documents will be retained for a longer period if required by the applicable regulatory requirements or by an agreement with the sponsor. It is the responsibility of the sponsor to inform the investigator/institution as to when these documents no longer need to be retained. If the responsible investigator retires, relocates, or for other reasons withdraws from the responsibility of keeping the study records, custody must be transferred to a person who will accept the responsibility. The sponsor must be notified in writing of the name and address of the new custodian. Under no circumstance shall the investigator relocate or dispose of any study documents before having obtained written approval from the sponsor. If it becomes necessary for the sponsor or the appropriate regulatory authority to review any documentation relating to this study, the investigator/institution must permit access to such reports.

10.9 Monitoring

The sponsor will perform on-site monitoring visits as frequently as necessary. The monitor will record dates of the visits in a study site visit log that will be kept at the study site. The first post-initiation visit will be made as soon as possible after enrollment has begun. At these visits, the monitor will compare the data entered into the CRFs with the hospital or clinic records (source documents). The nature and location of all source documents will be identified to ensure that all sources of original data required to complete the CRF are known to the sponsor and study-site personnel and are accessible for verification by the sponsor study-site contact. If electronic records are maintained at the study site, the method of verification must be discussed with the study-site personnel. Direct access to source documentation (medical records) must be allowed for the purpose of verifying that the data recorded in the CRF are consistent with the original source data. Findings from this review of CRFs and source documents will be discussed with the study-site personnel. The sponsor expects that, during monitoring visits, the relevant study-site personnel will be available, the source documentation will be accessible, and a suitable environment will be provided for

review of study-related documents. The monitor will meet with the investigator on a regular basis during the study to provide feedback on the study conduct.

10.10 Study Termination

The sponsor reserves the right to close the study site or terminate the study at any time for any reason at the sole discretion of the sponsor. Study sites will be closed upon study completion. A study site is considered closed when all required documents and study supplies have been collected and a study-site closure visit has been performed.
The investigator may initiate study-site closure at any time, provided there is reasonable cause and sufficient notice is given in advance of the intended termination.
Reasons for the early closure of a study site by the sponsor or investigator may include but are not limited to:

- Failure of the investigator to comply with the protocol, the requirements of the IEC/IRB or local health authorities, the sponsor's procedures, or GCP guidelines
- Inadequate recruitment of subjects by the investigator
- Discontinuation of further study drug development

10.11 On-Site Audits

Representatives of the sponsor's clinical quality assurance department may visit the study site at any time during or after completion of the study to conduct an audit of the study in compliance with regulatory guidelines and company policy. These audits will require access to all study records, including source documents, for inspection and comparison with the CRFs. Subject privacy must, however, be respected. The investigator and study-site personnel are responsible for being present and available for consultation during routinely scheduled study-site audit visits conducted by the sponsor or its designees. Similar auditing procedures may also be conducted by agents of any regulatory body, either as part of a national GCP compliance program or to review the results of this study in support of a regulatory submission. The investigator should immediately notify the sponsor if they have been contacted by a regulatory agency concerning an upcoming inspection.

10.12 Annual progress report

The sponsor/investigator will submit a summary of the progress of the trial to the accredited METC once a year. Information will be provided on the date of inclusion of the first subject, numbers of subjects included and numbers of subjects that have completed the trial, serious adverse events/serious adverse reactions, other problems, and amendments.

10.13 End of study report

The investigator will notify the accredited METC of the end of the study within a period of 10 weeks. The study is considered completed with the last study assessment for the last subject participating in the study. The final data from the study site will be sent to the sponsor (or designee) after completion of the final subject assessment at that study site, in the time frame specified in the Clinical Trial Agreement. Within one year after the end of the study, the investigator/sponsor will submit a final study report with the results of the study, including any publications/abstracts of the study, to the accredited METC. In case the study is ended prematurely, the investigator will notify the accredited METC, including the reasons for the premature termination.

10.14 Public disclosure and publication policy

The results of this study will be published as soon as possible in international peer reviewed journals and national journals, in accordance with the sponsor. Herein described data can in no way be traced back to the participants. All information, including but not limited to information regarding Healseeker or the sponsor's operations (eg, patent application, formulas, manufacturing processes, basic scientific data, prior clinical data, formulation information) supplied by the sponsor to the investigator and not previously published, and any data, including research data, generated as a result of this study, are considered confidential and remain the sole property of the sponsor. The investigator agrees to maintain this information in confidence and use this information only to accomplish this study, and will not use it for other purposes without the sponsor's prior written consent. The investigator understands that the information developed in the study will be used by the sponsor in connection with the continued development of Healseeker, and thus may be disclosed as required to other clinical investigators or regulatory agencies. To permit the information derived from the clinical studies to be used, the investigator is obligated to provide the sponsor with all data obtained in the study. The results of the study will be reported in a Clinical Study Report generated by the sponsor and will contain CRF data from all study sites that participated in the study. Recruitment performance or specific expertise related to the nature and the key assessment parameters of the study will be used to determine a coordinating investigator. Results of study analyses performed after the Clinical Study Report has been issued will be reported in a separate report and will not require a revision of the Clinical Study Report. Study subject identifiers will not be used in publication of results. Any work created in connection with performance of the study and contained in the data that can benefit from copyright protection (except any publication by the investigator as provided for below) shall be the property of the sponsor as author and owner of copyright in such work. Consistent with Good Publication Practices and International Committee of Medical

Journal Editors guidelines, the sponsor shall have the right to publish such primary (multicenter) data and information without approval from the investigator. The investigator has the right to publish study site-specific data after the primary data are published. If an investigator wishes to publish information from the study, a copy of the manuscript must be provided to the sponsor for review at least 60 days before submission for publication or presentation. Expedited reviews will be arranged for abstracts, poster presentations, or other materials. If requested by the sponsor in writing, the investigator will withhold such publication for up to an additional 60 days to allow for filing of a patent application. In the event that issues arise regarding scientific integrity or regulatory compliance, the sponsor will review these issues with the investigator. The sponsor will not mandate modifications to scientific content and does not have the right to suppress information. For multicenter study designs and substudy approaches, secondary results generally should not be published before the primary endpoints of a study have been published. Similarly, investigators will recognize the integrity of a multicenter study by not submitting for publication data derived from the individual study site until the combined results from the completed study have been submitted for publication, within 12 months of the availability of the final data (tables, listings, graphs), or the sponsor confirms there will be no multicenter study publication. Authorship of publications resulting from this study will be based on the guidelines on authorship, such as those described in the Uniform Requirements for Manuscripts Submitted to Biomedical Journals, which state that the named authors must have made a significant contribution to the design of the study or analysis and interpretation of the data, provided critical review of the paper, and given final approval of the final version.

10.15 Registration of Clinical Studies and Disclosure of Results

The sponsor will register and/or disclose the existence of and the results of clinical studies as required by law.

11. STRUCTURED RISK ANALYSIS

11.1 Potential issues of concern

a. Level of knowledge about mechanism of action

Computer games are increasingly used within health care settings to educate its staff members. This study is innovative as it tries to change the behaviour of children with ADHD within a mental health care setting. The main mechanism of action is thought to be the ability of the computer game to increase the motivation of the player, thereby trying to reduce associated problems of ADHD and learning children strategies to cope with these difficulties. Increased motivation makes it possible to practice and learn new behaviour. Also the repetitive nature of computer games promotes learning of new behaviour. By playing a serious game children can learn while engaging in a pleasurable activity.

b. Previous exposure of human beings with the test product(s) and/or products with a similar biological mechanism

Not applicable.

c. Can the primary or secondary mechanism be induced in animals and/or in *ex-vivo* human cell material?

Not applicable.

d. Selectivity of the mechanism to target tissue in animals and/or human beings

Not applicable.

e. Analysis of potential effect

The results of the pilot study show that no adverse events occur when playing the computer game eight times in two weeks for a period of two months. Therefore no adverse effects are expected when playing the computer game three times per week. The children who played the game eight times per two weeks in the pilot study had better results on time management and planning at posttest measurements compared to pretest measurements.

f. Pharmacokinetic considerations

Not applicable.

g. Study population

This study will include a total of 170 children with an official DSM-IV ADHD diagnosis, randomly selected among the research sites in The Netherlands and Belgium. Children with

common diagnosed comorbid disorders (i.e. dyslexia, oppositional defiant disorder) can participate in the study. All children must be between 8 and 12 years of age. All participants need to be stable on ADHD treatment, both pharmacological and psychological, for at least two months prior to start of the intervention. Participants must, when possible, continue this treatment throughout the intervention period and between post-test and follow-up measurements. The children must have a minimum total intelligence score of 80. All children and one of the parent/legal guardians must have a reasonable understanding of the Dutch language in order to understand the messages in the game, and have clear communication with the investigators.

h. Interaction with other products

So far, no research has been performed to measure possible effects of the interaction between gaming and medication use.

i. Predictability of effect

As the goal of the intervention is to accomplish behavior change in children with ADHD most of the measurements are behavior questionnaires. The expertise of the investigators is needed to monitor the behavior and to interpret the test results of the participating children.

j. Can effects be managed?

All participants receive a patient card with the contact information of the investigators and the independent professional, whom they can contact in case of emergency or questions about the study.

11.2 Synthesis

Based on the above mentioned risk taxation (and paragraph 9.4) we presume participating in the study is without any risk. The risks of participation can be considered negligible and the burden can be considered minimal. The computer game can be played online and at home. The time to play the game is restricted and set at 45 minutes per game moment. The goal of this training is to enhance time management, planning and organisational skills and social skills in children with ADHD through a computer game with built-in learning goals. This study provides children with ADHD with the opportunity to join a non-medicinal intervention aimed at abating associated problems and elevating empowerment. Parents/legal guardians and the child can withdraw from the intervention at any moment, without questions or further consequences.

12. REFERENCES

Abikoff, H., Nisseley-Tsiopinis, J., Gallagher, R., Zambenedetti, M., Seyffert, M., Boodray, R. & McCarthy, J. (2009). Effects of MPH-OROS on the organizational, time management and planning behaviors of children with ADHD. *Journal of the American Academy of Child & Adolescent Psychiatry, 48,* 166-175.

Featured Free Online ADHD games (video game file). Retrieved from http://www.fupa.com games/1/adhd.html

Alloway, T.P. Gathercole, S.E., & Pickering, S.J. (2006). Verbal and visuospatial short-term and working memory in children: Are they separable? *Child Development, 77*(6), 1698–1716. doi: 10.1111/j.1467-8624.2006.00968.x

Antshel, K. & Remer, R. (2003). Social skills training in children with Attention Deficit Hyperactivity Disorder: A randomized-controlled clinical trial. *Journal of Clinical Child & Adolescent Psychology, 23*(1), 153-165. doi: 10.1207/S15374424JCCP3201_14

Amon, K. L. & Campbell, A. (2008). Can children with AD/HD learn relaxation and breathing techniques through biofeedback games? *Australian Journal of Educational & Developmental Psychology, 8,* 72-84.

Bandura, A. (2006). Toward a psychology of human agency. *Perspectives on Psychological Science, 1*(2), 164-180. doi: 10.1111/j.1745-6916.2006.00011.x

Barkley, R. A. (1998). It's about time. Unpublished.

Barkley, R. A. (2006). *Attention-deficit hyperactivity disorder. A handbook for diagnosis and treatment. 3rd ed.* New York: Guilford Press.

Beck, S.J., Hanson, C.A., Puffenberger. S.S., Beninger, K.L., & Benninger, W.B. (2010). A controlled trial of working memory training for children and adolescents with ADHD. *Journal of Clinical Child & Adolescent Psychology, 39*(6), 825-836. doi: 10.1080/15374416.2010.517162

Bureau NMG (2003). *Nationale Monitor Geestelijke Gezondheid*. Retrieved from: http://www.trimbos.nl/

Chronis, A. M., Jones, H. A., & Raggi, V. L. (2006). Evidence-based psychosocial treatments for children and adolescents with attention-deficit/hyperactivity disorder. *Clinical Psychology Review, 26,* 486-502. doi: 10.1016/j.cpr.2006.01.002

Cohen, J. (1992). "A power primer". *Psychological Bulletin, 112* (1), 155–159. doi: 10.1037/0033-2909.112.1.155

Corsi, P. M. (1972). Human memory and the medial temporal region of the brain. *Dissertation Abstracts International, 34*(02), 891B.

Douma, S.H. (2008). *De meerwaarde van serious computergames en virtual riality bij de preventie en behandeling van psychische klachten.* Retrieved from umcg.wewi.eldoc.ub.rug.nl/root/Rapporten/2008/UBAssertiv4/

Dovis, S., Van der Oord, S., Wiers, R. W., & Prins, P. J. M. (2011). Can motivation normalize working memory and task persistence in children with Attention-Deficit/Hyperactivity Disorder? *The effects of money and computer-gaming, Journal of Abnormal Child Psychology, 40*(5), 669-681. doi: 10.1007/s10802-011-9601-8

Emes, C. (1997). Is Mr Pac Man eating our children: A review of the effects of videogames on children. *The Canadian Journal of Psychiatry, 42*(4), 409-414.

Gevensleben, H., Holl, B., Albrecht, B., Schlamp, D., Kratz, O., Studer, P., Rothenberger, A., Moll, G.H., & Heinrich, H. (2010). Neurofeedback training in children with ADHD: 6-month follow-up of a randomised controlled trial. *European Child & Adolescent Psychiatry, 19*(9), 715-24.doi: 10.1007/s00787-010-0109-5

Gibson, B.S., Gondoli, D.M., Johnson, A.C., Steeger, C.M., Dobrzenski, B.A., & Morrisey, R.A. (2011). Component analysis of verbal versus spatial working memory training in adolescents with ADHD: a randomized, controlled trial. Child Neuropsychology, 17(6), 546-63. doi: 10.1080/09297049.2010.551186

Gresham, M. & Elliott, S. N. (2008). *Rating Scales Manual. SSIS Social Skills Improvement System.* Minneapolis, Pearson.

Hochberg (1988) A sharper Bonferroni procedure for multiple tests of significance. Biometrika 75(4):800-802

Kane, A. (2007). *Is your child rejected by other children?* Retrieved from http://www.disabled-world.com/artman/publish/adhd-child-rejection.shtml.

Kato, P.M., Cole, S.W., Bradlyn, A.S., & Pollock, B.H. (2008). A video game improves behavioral outcomes in adolescents and young adults with cancer: A randomized trial. *Pediatrics, 122*(2), 305-317. doi: 10.1542/peds.2007-3134

Kats-Gold, I., & Priel, B. (2009). Emotion, understanding, and social skills among boys at risk of attention deficit hyperactivity disorder. *Psychology in the Schools, 46*(7), 658-678. doi: 10.1002/pits.20406

Kofler, M.J., Rapport, M.D., Bolden, J., Sarver, D.E., Raiker, J.S., & Alderson, R.M. (2011). Working memory deficits and social problems in children with ADHD. *Journal of Abnormal Child Psychology, 39*(6), 805–817. doi: 10.1007/s10802-011-9492-8

Kohlberg, J., & Nadeau, K. (2007). *Opgeruimd leven met ADHD. Praktische organiseertips- en strategieën*,1th ed. Amsterdam: Nieuwezijds B.V.

Kranenburg, K., Slot, M., Staal, M., Leurdijk, A., & Burgmeijer, J. (2006). *Serious Gaming. Onderzoek naar knelpunten en mogelijkheden van serious gaming.* Delft: TNO.

Martin, B.D. (2009). A qualitative study of social skill learning opportunities in massively multiplayer online role-playing games. *Dissertation Abstracts International Section A: Humanities and Social Sciences, 69*(11), 4530.

Martinussen, R., Hayden, J., Hogg-Johnson, S., & Tannock, R. (2005). A meta-analysis of working memory impairments in children with Attention-Deficit/Hyperactivity Disorder. *Journal of the American Academy of Child and Adolescent Psychiatry, 44*(4), 377–384. doi: 10.1097/01.chi.0000153228.72591.73

Melby-Lervåg, M., & Hulme, C. (2012). Is Working Memory Training Effective? A Meta-Analytic Review. *Developmental Psychology*. Advance online publication. doi: 10.1037/a0028228

Melnick, S. M., & Hinshaw, S. P. (2000). Emotion regulation and parenting in ADHD and comparison boys: Linkages with social behaviors and peer preference. *Journal of Abnormal Child Psychology, 28*(1), 73-86. doi: 10.1023/A:1005174102794

Mikami, A. Y. (2010). The importance of friendship for youth with Attention-Deficit/Hyperactivity Disorder. *Clinical Child and Family Psychology Review, 13*(2) 181-198. doi: 10.1007/s10567-010-0067-y

MTA Cooperative Group (1999). A 14-month randomized clinical trial of treatment strategies for ADHD. *Archives of General Psychiatry*, *56*(12), 1073 –1086.

Nair, J., Ehimar, U., Beitman, B. D., Nair, S. S. & Lavin, A. (2006). Clinical review: Evidence-based diagnosis and treatment of ADHD in children. *Missouri Medicine, 103*(6) 617-621.

NJI kenniscentrum. (2008) *Prevalentie van aandachtsstoornissen met hyperactiviteit.* Retrieved from http://www.nji.nl/

Oosterlaan, J., Baeyens., D. Scheres, A., Antrop, I., Roeyers, H. & Sergeant, J.A. (2008). *Vragenlijst voor gedragsproblemen bij kinderen 6-16 jaar, Handleiding.* Amsterdam: Harcourt Publishers.

Pelham, W.E., Gnagy, E.M., Greenslade, K.E., & Milich, R. (1992). Teacher ratings of DSM-III-R symptoms for disruptive behavior disorders. *Journal of the American Academy of Child and Adolescent Psychiatry, 31*, 210-218.

Pelham, W. E., Carlson, C., Sams, S. E., Vallano, G., Dixon, M. J., & Hoza, B. (1993). Seperate and combined effects of methylfenidate and behavior modification on the classroom behavior and academic performance of ADHD boys: Group effects and individual differences. *Journal of Consulting and Clinical Psychology, 61*(3), 506-515. doi: 10.1037/0022-006X.61.3.506

Peng, W., Lee, M., & Heeter, C. (2010). The effects of a serious game on role taking and willingness to help. *Journal of communication, 60*(4), 732-742. doi: 10.1111/j.1460-2466.2010.01511.x

Pfiffner, L., DuPaul, G. J. , Barkley, R. (2006). *Treatment of ADHD in school settings. In Barkley RA, ed. Handbook of attention deficit hyperactivity disorder, 3rd ed*. New York: Guilford Press.

Polanczyk, G., de Lima, M. S., Horta, B. L., Biederman, J. & Rohde, L. A. (2007). The worldwide prevalence of ADHD: A systematic review and metaregression analysis. *American Journal of Psychiatry*, *164*(6), 942-948.

Pope, A.T., & Bogart, E.H. (1996). Extended attention span training system: Video game neurotherapy for Attention Deficit Disorder. *Child Study Journal, 26*(1), 39-50.

Prins, P. J. M., Dovis, S., Ponsioen, A., Ten Brink, E., & Van der Oord, S. (2011). Does computerized working memory training with game elements enhance motivation and training efficacy in children with ADHD? *Cyberpsychology, Behavior and Social Networking, 14,* 115-122. doi:10.1089/cyber.2009.0206.

Puig-Antich, J., & Chamber, W. (1978). *The Schedule for Affective Disorders and Schizophrenia for school –Age Children (Kiddie-SADS).* New York: New York State Psychiatric Institute.

Reichart, C.G., Wals, M., & Hillegers, M. (2000). *Vertaling K-sads.* Utrecht: H.C. RümkeGroep.

Rosas, R., Ceric, F., Tenorio, M., Mourgues, C., Thibaut, C., Hurtado, E., & Aravena, M. T. (2010). ADHD children outperform normal children in an artificial grammar implicit learning task: ERP and RT evidence. *Consciousness and Cognition, 19*(1) 341-351. doi: 10.1016/j.concog.2009.09.006

Sattler, J. M. (2001). *Assessment of children: Cognitive applications* (4th ed.). San Diego: Author.

Schachter, H. M., Pham, B., King, J., Langford, S., & Moher, D. (2001). How efficacious and safe is short-acting methylphenidate for the treatment of attention-deficit hyperactivity disorder in children and adolescents? A meta-analysis. *Canadian Medical Association Journal,* 165(11), 1475-1488.

Smidts, D.P. & Huizinga, M. (2009). *BRIEF Executieve Functies Gedragsvragenlijst: Handleiding*. Amsterdam: Hogrefe Uitgevers.

Schmeichel, B.J. (2010). Working memory capacity and spontaneous emotion regulation: high capacity predicts self-enhancement in response to negative feedback. *Emotion, 10*(5), 739-744. doi: 10.1037/a0019355

Spence, S. H. (2003). Social skills training with children and young people: Theory, evidence and practice. *Child and Adolescent Mental Health, 8*(2), 84–96. doi: 10.1111/1475-3588.00051

Tannock, R. (1997). Television, videogames, and ADHD: Challenging a popular belief. *The ADHD Report, 5,* 3-7.

Unterrainer,J.M., Rahm, B., Kaller, C.P., Leonhart, R., Quiske, K., Hoppe-Seyler, K., Meier, C., Muller, C., & Halsband, U. (2004). Planning abilities and the Tower of London: is this task measuring a discrete cognitive function? *Journal of Clinical and Experimental Neuropsychology*, *26*(6), 846-56.

Van der Oord, S. V., Ponsioen, A. J., Geurts, H. M., Ten Brink E., & Prins, P, J. (*2012*). Pilot study of the efficacy of a computerized executive functioning remediation training with game elements for children with ADHD in an outpatient setting: Outcome of parent- and teacher-rated executive functioning and ADHD behavior. *Journal of Attention Disorders.* doi:10.1177/1087054712453167

Van der Oord, S. V., Prins, P.J.M., Oosterlaan, J., & Emmelkamp, P.M.G. (2008). Efficacy of methylphenidate, psychosocial treatments and their combination in school-aged children with ADHD: A meta-analysis. *Clinical Psychology Review, 28* (5), 783-800. doi: 10.1016/j.cpr.2007.10.007

Van Manen, T. G., Prins, P. J. M., & Emmelkamp, P. M. G. (2007). *Handleiding Sociaal Cognitieve Vaardigheden Test.* Houten: Bohn Stafleu van Loghum.

Van Rooij, A. J., Schoenmakers, T. M., Meerkerk, G. J., & Van de Mheen, D. (2009). *Introduction to video games, their publishers, and social responsibility concerning video game addiction*. Rotterdam: IVO. Retrieved from http://bit.ly/da1uvR

Vink, M., & Van Wamel, A. (2007). *Landelijk ADHD programma bij kinderen en jeugdigen.* Utrecht: Trimbos-Instituut.

Wechsler, D. (2000). *WAIS-III Nederlandstalige Bewerking.* Technische Handleiding. Lisse: Swets & Zeitlinger.

Wechsler, D. (2005). *Wechsler Intelligence Scale for Children-Third Edition, Nederlandse Vertaling (WISC-III-NL)*. Lisse: Harcourt Test Publishers.

Wender, P.H., Wolf, L.E., & Wasserstein, J. (2001). Adults with ADHD. An overview. *Annals of the New York Academy of Sciences, 931*, 1-16. doi: 10.1111/j.1749-6632.2001.tb05770.x

13. Attachments

A. Severity of symptoms (VvGK 6-16)

Het is de bedoeling dat u bij onderstaande beschrijvingen (middels helemaal niet, een beetje, tamelijk veel, heel veel) aangeeft in welke mate deze van toepassing is op uw kind zoals hij/zij nu is of in de afgelopen twee weken is geweest.

		helemaal niet	een beetje	tamelijk veel	heel veel
1	Valt anderen in de rede of dringt zich vaak op (bijvoorbeeld: mengt zich zomaar in gesprekken of spelletjes)	☐	☐	☐	☐
2	Is tenminste tweemaal van huis weggelopen en 's nachts weggebleven terwijl hij / zij thuis of in een gezinsvervangend huis woonde (of eenmaal gedurende een langere periode zonder terug te komen)	☐	☐	☐	☐
3	Maakt ruzie met volwassenen	☐	☐	☐	☐
4	Liegt om goederen of gunsten te krijgen of om verplichtingen uit de weg te gaan (bijvoorbeeld: licht anderen op)	☐	☐	☐	☐
5	Begint vechtpartijen met huisgenoten	☐	☐	☐	☐
6	Mishandelt mensen lichamelijk	☐	☐	☐	☐
7	Praat aan één stuk door	☐	☐	☐	☐
8	Heeft voorwerpen van niet onbeduidende waarde gestolen zonder direct contact met het slachtoffer (bijvoorbeeld: winkeldiefstal, maar zonder in te breken; vervalsing)	☐	☐	☐	☐
9	Wordt gemakkelijk afgeleid door prikkels van buitenaf	☐	☐	☐	☐
10	Spijbelt en doet dit al vóór zijn / haar dertiende jaar	☐	☐	☐	☐
11	Beweegt onrustig met handen of voeten, of zit op de stoel te draaien	☐	☐	☐	☐
12	Is hatelijk of wraakzuchtig	☐	☐	☐	☐
13	Geeft anderen de schuld van eigen fouten of wangedrag	☐	☐	☐	☐
14	Heeft opzettelijk eigendommen van anderen vernield (hieronder valt niet brandstichting)	☐	☐	☐	☐
15	Verzet zich tegen of weigert zich te voegen naar verzoeken of regels van volwassenen	☐	☐	☐	☐
16	Lijkt niet te luisteren als hij / zij direct aangesproken wordt	☐	☐	☐	☐
17	Flapt het antwoord er al uit nog voordat de vraag goed en wel gesteld is	☐	☐	☐	☐
18	Begint vechtpartijen met anderen die niet bij hem / haar in huis wonen (bijvoorbeeld: leeftijdgenootjes op school of in de buurt)	☐	☐	☐	☐
19	Heeft er moeite mee om rustig te spelen of zichzelf tijdens vrije tijd rustig te vermaken	☐	☐	☐	☐
20	Schenkt onvoldoende aandacht aan details of maakt slordigheidsfouten in het schoolwerk, in het werk, of tijdens andere activiteiten	☐	☐	☐	☐
21	Is vaak boos en verontwaardigd	☐	☐	☐	☐

		helemaal niet	een beetje	tamelijk veel	heel veel
22	Gaat vaak van zijn / haar stoel af in de klas of in andere situaties waar verwacht wordt dat men blijft zitten				
23	Is vaak prikkelbaar of ergert zich gemakkelijk aan anderen				
24	Geeft vaak geen gehoor aan aanwijzingen en slaagt er vaak niet in schoolwerk, karweitjes, of verplichtingen op het werk af te maken (niet als gevolg van tegendraads gedrag of het niet begrijpen van aanwijzingen)				
25	Wordt vaak boos				
26	Heeft vaak moeite de aandacht vol te houden bij taken of spel				
27	Heeft vaak moeite op zijn / haar beurt te wachten				
28	Heeft iemand tot seksuele activiteiten gedwongen				
29	Terroriseert, bedreigt of intimideert vaak anderen				
30	Is vaak in de weer of draaft maar door				
31	Raakt vaak dingen kwijt die nodig zijn voor taken of activiteiten (bijvoorbeeld: speelgoed, huiswerk, potloden, boeken of gereedschap)				
32	Rent vaak rond of klimt overal op in situaties waarin dit ongepast is (bij adolescenten of volwassenen kan dit beperkt zijn tot subjectieve gevoelens van rusteloosheid)				
33	Heeft dieren mishandeld				
34	Vermijdt vaak, heeft een hekel aan, of is onwillig zich bezig te houden met taken die een langdurige geestelijke inspanning vereisen (zoals school- of huiswerk)				
35	Blijft vaak, ondanks het verbod van de ouders, 's nachts van huis weg, beginnend vóór het dertiende jaar				
36	Ergert vaak met opzet anderen				
37	Heeft iemand bestolen (bijvoorbeeld: iemand aanvallen en beroven, tasjesroof, afpersing, gewapende overval)				
38	Heeft opzettelijk brand gesticht met de bedoeling ernstige schade aan te richten				
39	Heeft vaak moeite met het organiseren van taken en activiteiten				
40	Heeft ingebroken in andermans huis, gebouw of auto				
41	Is vaak vergeetachtig bij dagelijkse bezigheden				
42	Heeft een wapen gebruikt dat anderen ernstig lichamelijk letsel kan toebrengen (bijvoorbeeld: een knuppel, steen, gebroken fles, mes, vuurwapen)				

B. General Questionnaire Parents, version 02

Onderstaande vragen hebben betrekking op algemene gegevens van u en uw kind. Er wordt aan u gevraagd uit één alternatief te kiezen en het bijbehorende antwoord aan te kruisen. Bij enkele andere vragen kunt u het antwoord achter de vraag schrijven.

Achtergrondgegevens moeder

1. Werkt moeder (neemt deel aan arbeidsproces)?

- ☐ voltijd
- ☐ deeltijd
- ☐ niet
- ☐ niet van toepassing
- ☐ Anders, nl___________________________

2. Beroep moeder: ____

Achtergrond gegevens vader

3. Werkt vader (neemt deel aan arbeidsproces)?

- ☐ voltijd
- ☐ deeltijd
- ☐ niet
- ☐ niet van toepassing
- ☐ Anders, nl___________________________

4. Beroep vader: ____

5. Welke taal wordt er thuis het meest gesproken?

- ☐ Nederlands
- ☐ Vlaams
- ☐ Frans
- ☐ Anders, nl__________________________

Achtergrondgegevens school

6.

Naam: ____

Straat en huisnummer: ____

Plaats:____

Telefoonnummer: ____

Voor- en achternaam leerkracht: _____________________________________

E-mail leerkracht (indien bekend):____________________________________

7. Volgt uw kind speciaal onderwijs?

☐ ja ☐ nee

8. In welke groep/leerjaar zit uw kind op dit moment?

Groep/leerjaar ______

9. Is uw kind ooit blijven zitten?

☐ ja ☐ nee

10. Heeft uw kind een klas overgeslagen?

☐ ja ☐ nee

Medische achtergrond van uw kind

11. Gebruikt uw kind momenteel medicatie voor ADHD?

☐ ja ☐ nee

12. Wordt uw kind momenteel psychiatrisch of psychotherapeutisch behandeld voor ADHD?

☐ ja ☐ nee

13. Ontvangt u momenteel ouderbegeleiding vanuit de eigen GGZ-instelling?

☐ ja ☐ nee

14. Ontvangt u momenteel psycho-educatie vanuit de eigen GGZ-instelling?

☐ ja ☐ nee

C. BRIEF Parent version, Subscale Planning and Organizing, version 01

Het is de bedoeling dat u bij onderstaande beschrijvingen (middels nooit, soms, vaak) aangeeft in welke mate deze van toepassing is op uw kind zoals hij/zij nu is of in de afgelopen twee weken is geweest.

	Nooit	Soms	Vaak
1. Brengt huiswerk, opgaven, spulletjes etc. niet mee naar huis	○	○	○
2. Heeft goede ideeën, maar krijgt ze niet op papier	○	○	○
3. Legt geen link tussen het doen van huiswerk en het behalen van cijfers (als uw kind nooit huiswerk heeft, kruis hier het vakje “nooit” aan)	○	○	○
4. Vergeet het huiswerk in te leveren, ook als het af is (als uw kind nooit huiswerk heeft, kruis hier het vakje “nooit” aan)	○	○	○
5. Raakt verstrikt in details en verliest het algemene overzicht	○	○	○
6. Heeft goede ideeën, maar kan ze niet uitvoeren (geen doorzettingsvermogen)	○	○	○
7. Raakt overweldigd door grote opgaven	○	○	○
8. Onderschat de tijd die nodig is om taken af te maken	○	○	○
9. Begint pas op het laatste moment aan opdrachten of karweitjes	○	○	○
10. Denkt niet vooruit bij huiswerkopdrachten (als uw kind nooit huiswerk heeft, kruis hier het vakje “nooit” aan)	○	○	○
11. Geschreven werk ziet er slordig uit	○	○	○
12. Heeft moeite dingen te doen die nodig zijn om doelen te bereiken (geld sparen voor iets speciaals, leren voor een goed cijfer)	○	○	○

D. Time management self constructed questionnaire, version 01

Het is de bedoeling dat u bij onderstaande beschrijvingen (middels een cijfer van 1 tot en met 10) aangeeft in welke mate deze van toepassing is op uw kind zoals hij/zij nu is of in de afgelopen twee weken is geweest.

	Helemaal niet waar									Helemaal waar
	**1**	**2**	**3**	**4**	**5**	**6**	**7**	**8**	**9**	**10**
1. Mijn kind kan een korte taak afmaken, binnen de door u bepaalde tijd	○	○	○	○	○	○	○	○	○	○
2. Mijn kind kan bedenken hoeveel tijd nodig is om een klusje voor een deadline af te maken	○	○	○	○	○	○	○	○	○	○
3. Mijn kind kan zonder hulp binnen een bepaalde tijd het ochtendritueel doorlopen	○	○	○	○	○	○	○	○	○	○
4. Mijn kind kan zonder hulp binnen een redelijke tijd dagelijkse activiteiten uitvoeren	○	○	○	○	○	○	○	○	○	○
5. Mijn kind kan zijn/haar huiswerkplanning aanpassen ten behoeve van andere activiteiten (bijvoorbeeld vroeger beginnen op een avond dat hij/zij gaat sporten of naar muziekles gaat)	○	○	○	○	○	○	○	○	○	○
6. Mijn kind kan op tijd met een lange termijnproject beginnen. Zodat de kans op tijdnood kleiner wordt	○	○	○	○	○	○	○	○	○	○
7. Mijn kind heeft huiswerk meestal voor bedtijd af	○	○	○	○	○	○	○	○	○	○
8. Mijn kind neemt goede beslissingen over prioriteiten als de tijd beperkt is (gaat bijvoorbeeld vanuit school direct naar huis om een lange termijnopdracht af te maken en gaat niet met vrienden spelen)	○	○	○	○	○	○	○	○	○	○
9. Mijn kind kan een lange termijnproject over verschillende dagen uitsmeren	○	○	○	○	○	○	○	○	○	○
10. Mijn kind raffelt op het laatste moment klusjes en/of taken af	○	○	○	○	○	○	○	○	○	○
11. Mijn kind kijkt uit zichzelf regelmatig op de klok (of zijn/haar horloge)	○	○	○	○	○	○	○	○	○	○

E. "It is about time" Questionnaire, Parent version, Revision 17.02.1998

Het is de bedoeling dat u bij onderstaande beschrijvingen (middels zelden, soms, meestal, bijna altijd) aangeeft in welke mate deze van toepassing is op uw kind zoals hij/zij nu is of in de afgelopen twee weken is geweest.

	Zelden	Soms	Meestal	Bijna altijd
U geeft uw kind een taak of opdracht die hij/zij binnen een bepaalde tijdslimiet moet uitvoeren. Hoe groot is de kans dat hij/zij erin slaagt om die taak af te ronden binnen de tijdslimiet?	○	○	○	○
Hoe vaak praat uw kind over wat hij/zij heeft gedaan in het verleden in vergelijking met andere kinderen van dezelfde leeftijd?	○	○	○	○
Hoe vaak stelt uw kind vragen over zijn/haar verleden?	○	○	○	○
Hoe vaak praat uw kind over toekomstige of naderende gebeurtenissen waarbij hij/zij betrokken zal zijn?	○	○	○	○
Hoe vaak stelt uw kind vragen over dingen die zullen gebeuren in de toekomst?	○	○	○	○
Hoe vaak gebruikt uw kind een klok of horloge om na te gaan hoeveel tijd hij/zij nog heeft om iets te doen?	○	○	○	○
Hoe vaak plant uw kind dingen met anderen die zullen plaatsvinden op een latere datum of een later tijdstip?	○	○	○	○
Uw kind moet een deadline respecteren. Hoe groot is de kans dat hij/zij klaar of voorbereid is tegen die deadline?	○	○	○	○
Het is bedtijd en uw kind ligt in bed. Hoe vaak praat of denkt uw kind na over wat hij/zij die dag heeft meegemaakt?	○	○	○	○
Het is bedtijd. Hoe vaak praat of denkt uw kind na over wat hij/zij de volgende dag zal doen?	○	○	○	○
Hoe vaak vindt uw kind het moeilijk om te wachten tot hij/zij iets mag doen wat hij/zij liefst meteen al zou doen?	○	○	○	○
Hoe vaak lijkt uw kind terug te denken in de tijd of kennis uit het verleden te gebruiken voor hij/zij reageert in een situatie?	○	○	○	○
Hoe vaak lijkt uw kind vooruit te denken of vooruitziend te zijn voor hij/zij reageert op een situatie?	○	○	○	○

	Zelden	Soms	Meestal	Bijna altijd
Hoe vaak lijkt uw kind vooraf te plannen wat hij/zij wil doen in vergelijking met andere kinderen van dezelfde leeftijd?	○	○	○	○
Hoe vaak heeft uw kind zijn/haar huiswerk op tijd klaar?	○	○	○	○
Hoe vaak is uw kind 's ochtends op schooldagen tijdig klaar om te vertrekken?	○	○	○	○
Hoe vaak denkt uw kind na over de toekomstige gevolgen van zijn/haar handelingen voor zichzelf?	○	○	○	○
Hoe vaak denkt uw kind na over de toekomstige gevolgen van zijn/haar handelingen voor anderen?	○	○	○	○
Uw kind werkt aan een taak. Hoe vaak lijkt hij/zij de taak te kunnen afronden binnen de toegestane tijd?	○	○	○	○
Uw kind gaat ergens naartoe om iets te doen (bv. hij/zij gaat buiten spelen) en u zegt hem/haar dat hij/zij op een bepaald tijdstip terug thuis moet zijn. Hoe groot is de kans dat hij/zij op tijd terug is?	○	○	○	○
Uw kind heeft een taak. Hoe vaak stelt hij/zij die taak uit?	○	○	○	○
Uw kind belooft iets voor u te doen op een later tijdstip van de dag. Hoe groot is de kans dat hij/zij dat nog uit zichzelf weet zonder dat u hem/haar eraan moet herinneren?	○	○	○	○
U belooft uw kind een beloning in de verre toekomst wanneer hij/zij nu meteen iets voor u doet. Hoe groot is de kans dat hij/zij die taak meteen doet?	○	○	○	○

**Uw kind moet over een paar weken een schooltaak (bv. een boekbespreking of wetenschappelijk werk) inleveren. Hoelang op voorhand begint hij/zij zich voor te bereiden op die deadline?**		
a)	Hij/zij begint er meteen aan te werken	○
b)	Hij/zij wacht tot een week voor de deadline en begint er dan aan te werken	○
c)	Hij/zij wacht tot een paar dagen voor de deadline en begint er dan aan te werken	○
d)	Hij/zij wacht tot een dag voor de deadline en begint er dan aan te werken	○
e)	Hij/zij wacht tot de dag van de deadline en begint er dan aan te werken.	○
f)	Hij/zij bereidt zich helemaal niet voor	○

**Hoe goed is het tijdsgevoel van uw kind ontwikkeld, over het algemeen en in vergelijking met andere kinderen van zijn/haar leeftijd?**		
a)	Slecht	○
b)	Onder het gemiddelde	○
c)	Gemiddeld	○
d)	Boven het gemiddelde	○
e)	Ver boven het gemiddelde	○

F. Social Skills Improvement System (SSIS) Rating Scales, Parent version, (1998)

Het is de bedoeling dat u bij onderstaande beschrijvingen (middels nooit, soms, vaak) aangeeft in welke mate deze van toepassing is op uw kind zoals hij/zij nu is of in de afgelopen twee weken is geweest.

	Nooit	*Soms*	*Vaak*
1. Gaat thuis op een goede manier met vrije tijd om.	○	○	○
2. Houdt uit zichzelf zijn/haar kamer schoon en netjes.	○	○	○
3. Spreekt thuis met een normaal stemgeluid.	○	○	○
4. Neemt uit zichzelf deel aan groepsactiviteiten.	○	○	○
5. Stelt zich voor aan onbekende mensen, zonder hiertoe aangezet te hoeven worden	○	○	○
6. Reageert op een goede manier wanneer andere kinderen hem/haar duwen of slaan	○	○	○
7. Vraagt winkelpersoneel om informatie of hulp.	○	○	○
8. Luistert wanneer een volwassene een groep toespreekt.	○	○	○
9. Weigert beleefd onredelijke verzoeken van anderen.	○	○	○
10. Nodigt anderen thuis uit.	○	○	○
11. Feliciteert gezinsleden met prestaties.	○	○	○
12. Maakt gemakkelijk vrienden.	○	○	○
13. Heeft veel interesses.	○	○	○
14. Vermijdt situaties die problemen zouden kunnen geven.	○	○	○
15. Ruimt speelgoed of andere spullen in het huis op.	○	○	○
16. Biedt uit zichzelf hulp aan bij taken.	○	○	○
17. Reageert goed op kritiek.	○	○	○
18. Neemt op een goede manier de telefoon aan.	○	○	○
19. Helpt uit zichzelf met huishoudelijke taken.	○	○	○
20. Stelt op een goede manier huisregels die onrechtvaardig zouden kunnen zijn ter discussie	○	○	○
21. Probeert huishoudelijke taken zelf uit te voeren, voordat hij/zij om hulp vraagt	○	○	○
22. Beheerst woede bij ruzie met andere kinderen.	○	○	○
23. Wordt aardig gevonden door andere kinderen	○	○	○
24. Begint eerder zelf een gesprek dan te wachten tot anderen daarmee beginnen	○	○	○

	Nooit	*Soms*	*Vaak*
25. Beëindigt onenigheid met u op een rustige manier.	○	○	○
26. Beheerst woede in conflictsituaties met u.	○	○	○
27. Geeft complimentjes aan vriendjes of andere kinderen in het gezin.	○	○	○
28. Voert binnen een redelijke tijd huishoudelijke taken uit.	○	○	○
29. Vraagt toestemming om spullen van gezinsleden te gebruiken.	○	○	○
30. Heeft zelfvertrouwen in sociale situaties, zoals feestjes of groepsuitjes.	○	○	○
31. Vraagt toestemming om naar buiten te gaan.	○	○	○
32. Reageert op een goede manier op pesten van vriendjes, broertjes of zusjes.	○	○	○
33. Maakt goed gebruik van de tijd dat hij/zij moet wachten op uw hulp bij huiswerk of andere taken	○	○	○
34. Accepteert voorstellen van vriendjes om samen dingen te gaan doen.	○	○	○
35. Maakt gemakkelijk de overgang van de ene activiteit naar de andere.	○	○	○
36. Werkt uit zichzelf samen met gezinsleden.	○	○	○
37. Reageert goed op complimenten van vriendjes.	○	○	○
38. Meldt ongelukken bij de aangewezen personen.	○	○	○

G. BRIEF Parent Version, Subscale Working Memory, version 01

Het is de bedoeling dat u bij onderstaande beschrijvingen (middels nooit, soms, vaak) aangeeft in welke mate deze van toepassing is op uw kind zoals hij/zij nu is of in de afgelopen twee weken is geweest.

	Nooit	Soms	Vaak
1. Onthoudt alleen het eerste of het laatste als hij/zij drie dingen te doen krijgt	○	○	○
2. Kan zich maar kort concentreren	○	○	○
3. Heeft moeite zich te concentreren op karweitjes, schoolwerk etc.	○	○	○
4. Is snel afgeleid door geluid, activiteit, uitzicht etc.	○	○	○
5. Heeft moeite met karweitjes of taken die meer dan een stap vereisen	○	○	○
6. Heeft hulp nodig van een volwassene om bij de les te blijven	○	○	○
7. Vergeet wat hij/zij aan het doen was	○	○	○
8. Als hij/zij iets moet halen, vergeet hij/zij wat het ook alweer was	○	○	○
9. Heeft moeite om dingen af te maken (karweitjes, huiswerk)	○	○	○
10. Heeft moeite om dingen te onthouden, zelfs voor een paar minuten			

H. Self Efficacy children, Self constructed questionnaire, version 02

Hoe ik over mijzelf denk.

Maak eerst de oefenvraag, zodat je weet hoe je straks de echte vragen moet beantwoorden.

Oefenvraag

Als nu aan je gevraagd werd om dingen op te tillen die een verschillend gewicht hebben, hoe zeker ben je dat je de volgende gewichten kan optillen:

Geef aan hoe zeker je bent door het aanduiden van een getal tussen de 0 en de 10, zoals aangegeven op onderstaande schaal.

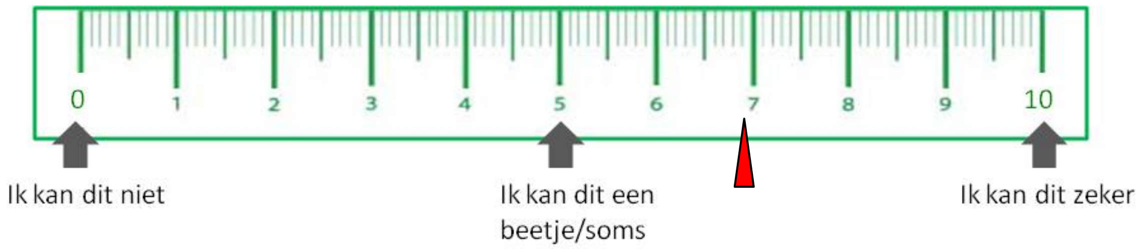

Optillen van een pak suiker

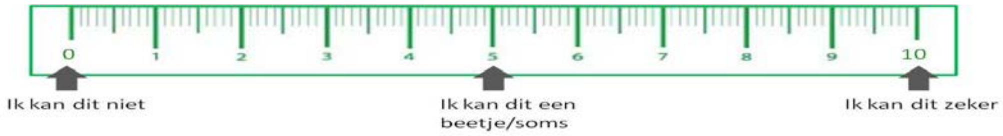

Een ingepakte koffer

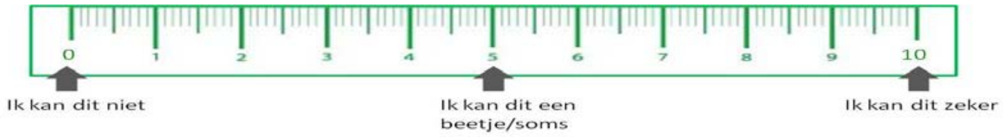

Een tafel

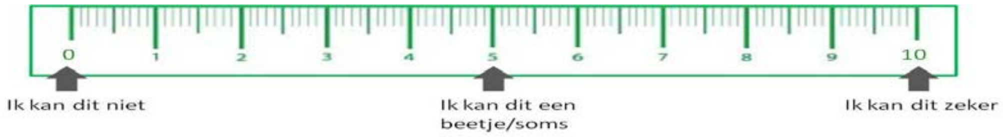

Een auto

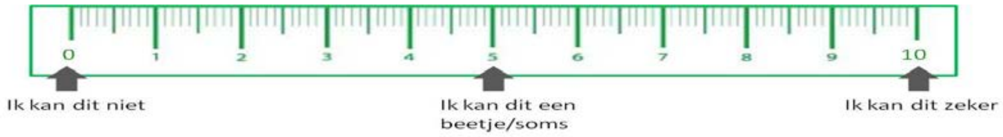

Vragenlijst voor kinderen

Deze vragenlijst is gemaakt, zodat wij beter begrijpen welke soort dingen kinderen moeilijk vinden om te doen.

Geef bij de volgende dingen aan hoe zeker je bent dat je deze dingen kan doen. Dit doe je door een nummer van 0-10 aan te duiden vullen.

1. Ik ben er zeker van dat ik naar een uitleg kan luisteren als ik haast heb.

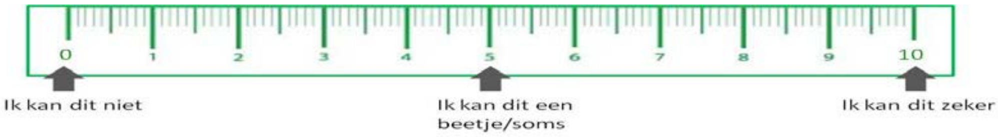

2. Ik ben er zeker van dat ik mijn taken kan plannen binnen een bepaalde tijd.

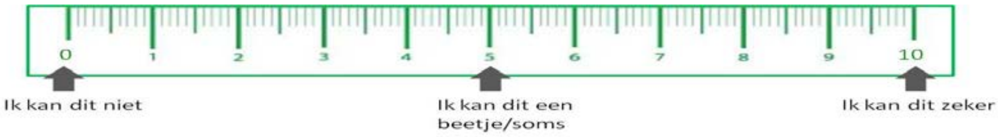

3. Ik ben er zeker van dat ik een vriend(in) of klasgenoot kan helpen, ook al heb ik zelf dingen die ik moet doen.

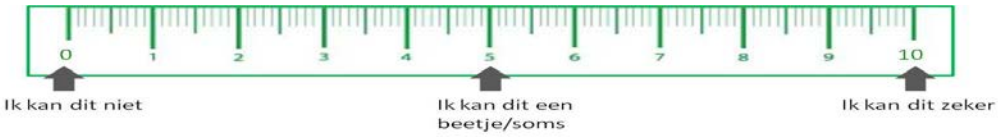

4. Ik ben er zeker van dat ik de tijd in de gaten kan houden door op een klok of horloge te kijken, zodat ik mijn taken op tijd af krijg.

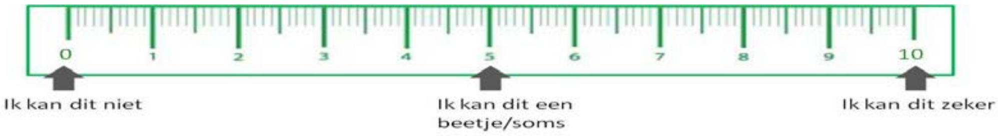

5. Ik ben er zeker van dat ik naar een uitleg kan luisteren, zonder er doorheen te praten.

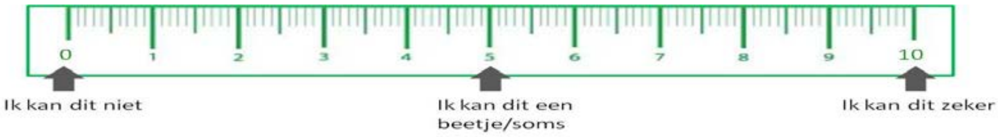

6. Ik ben er zeker van dat ik iets kan afmaken binnen de tijd die ik gepland had.

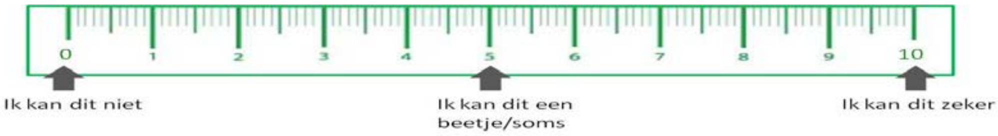

7. Ik ben er zeker van dat ik geconcentreerd kan blijven om taken te doen binnen mijn geplande tijd.

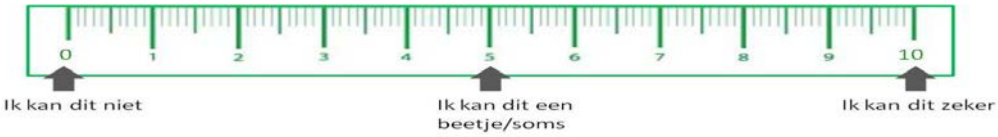

8. Ik ben er zeker van dat ik mijn taken doe binnen de tijd die ik gepland ervoor gepland heb.

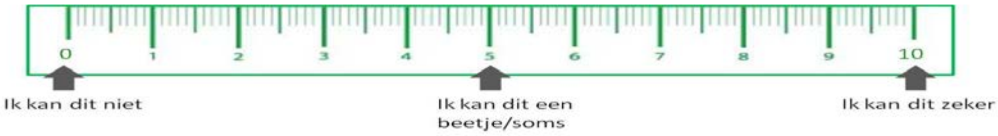

9. Ik ben er zeker van dat ik een nieuw plan kan proberen te maken, ook al is mijn eerste plan mislukt.

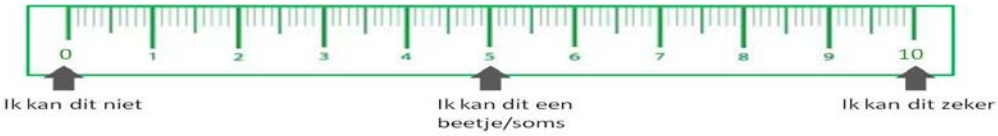

10. Ik ben er zeker van dat ik kan oefenen om beter te worden in het op tijd afmaken van taken.

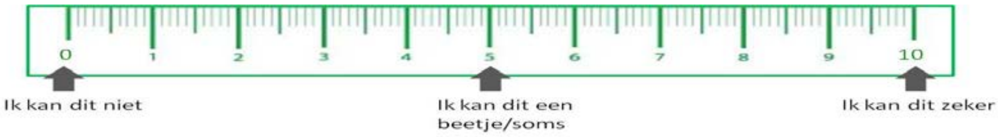

11. Ik ben er zeker van dat ik rustig kan blijven en kan doorzetten zelfs als mijn plan niet werkt.

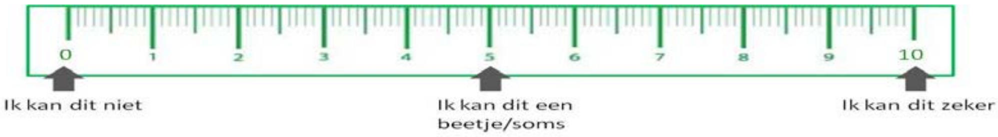

12. Ik ben er zeker van dat ik iets kan bereiken/afmaken zonder afgeleid of verveeld te raken.

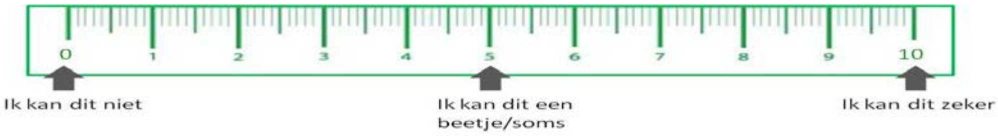

13. Ik ben er zeker van dat ik nieuwe manieren kan leren om taken op tijd af te krijgen.

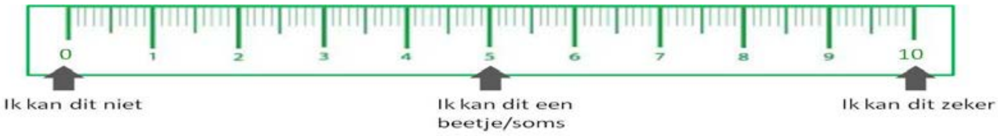

14. Ik ben er zeker van dat ik goede plannen kan blijven bedenken, zelfs als mijn plannen niet werken.

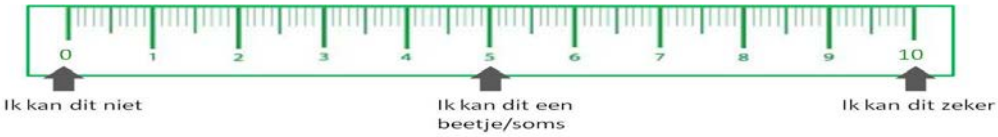

I. Questionnaires teachers, version 01

Het is de bedoeling dat u bij onderstaande beschrijvingen (middels nooit, soms, vaak) aangeeft in welke mate deze van toepassing is op uw leerling zoals hij/zij nu is of in de afgelopen twee weken is geweest.

	Nooit	Soms	Vaak
1. Onthoudt alleen het eerste of het laatste als hij/zij drie dingen te doen krijgt	○	○	○
2. Kan zich maar kort concentreren	○	○	○
3. Neemt huiswerk, opgaven, spulletjes etc. niet mee naar huis	○	○	○
4. Heeft goede ideeën, maar krijgt ze niet op papier	○	○	○
5. Heeft moeite zich te concentreren op karweitjes, schoolwerk etc	○	○	○
6. Is snel afgeleid door geluid, activiteit, uitzicht etc.	○	○	○
7. Vergeet huiswerk in te leveren, ook als het af is (als de leerling nooit huiswerk krijgt, kruis hier het vakje “nooit” aan)	○	○	○
8. Heeft moeite met karweitjes of taken die meer dan een stap vereisen	○	○	○
9. Heeft hulp nodig van een volwassene om bij de les te blijven	○	○	○
10. Raakt verstrikt in details en verliest het algemene overzicht	○	○	○
11. Vergeet wat hij/zij aan het doen was	○	○	○
12. Als hij/zij iets moet halen, vergeet hij/zij wat het ook alweer was	○	○	○
13. Heeft goede ideeën, maar kan ze niet uitvoeren (geen doorzettingsvermogen)	○	○	○
14. Raakt overweldigd door grote opgaven	○	○	○
15. Heeft moeite om dingen af te maken (karweitjes, huiswerk)	○	○	○
16. Onderschat de tijd die nodig is om taken af te maken	○	○	○
17. Begint pas op het laatste moment aan opdrachten of karweitjes	○	○	○
18. Denkt niet vooruit bij huiswerkopdrachten (als de leerling nooit huiswerk krijgt, kruis hier het vakje “nooit” aan)	○	○	○
19. Geschreven werk ziet er slordig uit	○	○	○
20. Heeft moeite om dingen te onthouden, zelfs voor een paar minuten	○	○	○
21. Beheerst zijn/haar woede in conflictsituaties met leeftijdsgenoten	○	○	○
22. Stelt zich uit zichzelf voor aan onbekenden.	○	○	○

	Nooit	Soms	Vaak
23. Stelt op een goede manier vragen bij regels die onrechtvaardig zouden kunnen zijn.	○	○	○
24. Past in conflictsituaties eigen mening aan om tot overeenstemming te komen.	○	○	○
25. Reageert op een goede manier op druk van leeftijdsgenoten.	○	○	○
26. Zegt op het juiste moment positieve dingen over zichzelf.	○	○	○
27. Nodigt anderen uit om aan activiteiten deel te nemen.	○	○	○
28. Gaat op een goede manier met vrije momenten om.	○	○	○
29. Voltooit opdrachten binnen de gegeven tijd.	○	○	○
30. Maakt gemakkelijk vrienden.	○	○	○
31. Reageert op een goede manier op pesten door leeftijdsgenoten.	○	○	○
32. Beheerst woede in conflictsituaties met volwassenen.	○	○	○
33. Gaat goed met kritiek om.	○	○	○
34. Begint uit zichzelf gesprekken met leeftijdsgenoten.	○	○	○
35. Maakt goed gebruik van de tijd terwijl hij/zij op hulp moet wachten.	○	○	○
36. Maakt zijn/haar schoolwerk netjes.	○	○	○
37. Vertelt u op een goede manier wanneer hij/zij vindt dat u hem/haar oneerlijk heeft behandeld.	○	○	○
38. Accepteert voorstellen van leeftijdsgenoten om samen dingen te doen.	○	○	○
39. Geeft complimenten aan leeftijdsgenoten.	○	○	○
40. Volgt uw instructies op.	○	○	○
41. Ruimt werkmateriaal of spullen van school op.	○	○	○
42. Werkt uit zichzelf samen met andere kinderen.	○	○	○
43. Biedt uit zichzelf aan om andere kinderen te helpen bij schooltaken.	○	○	○
44. Sluit zich aan bij een activiteit of een groepje zonder daartoe aangezet te worden.	○	○	○
45. Reageert op een goede manier bij duwen of slaan door andere kinderen.	○	○	○
46. Negeert storend gedrag van andere kinderen tijdens werken in de klas.	○	○	○
47. Houdt uit zichzelf zijn/haar tafel schoon en netjes.	○	○	○

	Nooit	Soms	Vaak
48. Luistert aandachtig wanneer u aanwijzingen geeft.	○	○	○
49. Maakt gemakkelijk de overgang van de ene klasactiviteit naar de andere.	○	○	○
50. Kan goed overweg met mensen die anders zijn.	○	○	○

Het is de bedoeling dat u bij onderstaande beschrijvingen (middels een cijfer van 1 tot en met 10) aangeeft in welke mate deze van toepassing is op uw leerling zoals hij/zij nu is of in de afgelopen twee weken is geweest.

	Helemaal niet waar									Helemaal waar
	**1**	**2**	**3**	**4**	**5**	**6**	**7**	**8**	**9**	**10**
1. Mijn leerling kan een korte taak afmaken, binnen de door u bepaalde tijd	○	○	○	○	○	○	○	○	○	○
2. Mijn leerling kan bedenken hoeveel tijd nodig is om een klusje voor een deadline af te maken	○	○	○	○	○	○	○	○	○	○
3. Mijn leerling kan zonder hulp binnen een bepaalde tijd het ochtendritueel doorlopen	○	○	○	○	○	○	○	○	○	○
4. Mijn leerling kan zonder hulp binnen een redelijke tijd dagelijkse activiteiten uitvoeren	○	○	○	○	○	○	○	○	○	○
5. Mijn leerling kan zijn/haar huiswerkplanning aanpassen ten behoeve van andere activiteiten	○	○	○	○	○	○	○	○	○	○
6. Mijn leerling kan op tijd met een lange termijnproject beginnen. Zodat de kans op tijdnood kleiner wordt	○	○	○	○	○	○	○	○	○	○
7. Mijn leerling heeft huiswerk meestal af	○	○	○	○	○	○	○	○	○	○
8. Mijn leerling neemt goede beslissingen over prioriteiten als de tijd beperkt is	○	○	○	○	○	○	○	○	○	○
9. Mijn leerling kan een lange termijnproject over verschillende dagen uitsmeren	○	○	○	○	○	○	○	○	○	○
10. Mijn leerling raffelt op het laatste moment klusjes en/of taken af	○	○	○	○	○	○	○	○	○	○
11. Mijn leerling kijkt uit zichzelf regelmatig op de klok (of zijn/haar horloge)	○	○	○	○	○	○	○	○	○	○

J. Game Experience Self constructed Questionnaire, version 01

Onderstaande vragen hebben betrekking op het computergebruik van uw kind. Er wordt aan u gevraagd uit één alternatief te kiezen en het bijbehorende antwoord aan te kruisen.

Vraag 1. Hoe vaak zit uw kind thuis achter de computer?

- ☐ nooit, want ik heb thuis geen computer
- ☐ nooit, maar ik heb thuis wel een computer
- ☐ ongeveer één keer per maand
- ☐ ongeveer één keer per week
- ☐ ongeveer twee tot drie keer per week
- ☐ (bijna) ieder dag

Vraag 2. Heeft u thuis een <u>spelcomputer</u> (o.a. xbox360, Nintendo, playstation)?

- ☐ nee
- ☐ ja

Vraag 3. Hoeveel uur per week speelt uw kind een spel op de computer/spelcomputer?

- ☐ 0 uur
- ☐ 1-5 uur
- ☐ 5-10 uur
- ☐ meer dan 10 uur

Vraag 4. Hoe ervaren vindt u uw kind op het gebied van computergebruik?

- ☐ beginner
- ☐ amateur
- ☐ ervaren
- ☐ expert

K. Satisfaction Parents, Self constructed questionnaire, version 02

Onderstaande vragen hebben betrekking op uw ervaringen met de verschillende aspecten van het computerspel. Het is de bedoeling dat u onderstaande vragen beantwoordt middels een cijfer van 1 tot en met 10. Bij enkele andere vragen kunt u het antwoord achter de vraag schrijven.

	Helemaal niet									Helemaal wel
	1	2	3	4	5	6	7	8	9	10
1. Hoe belastend was het spelen van het computerspel voor uw kind?	○	○	○	○	○	○	○	○	○	○

	Helemaal niet									Helemaal wel
	1	2	3	4	5	6	7	8	9	10
2. Hoe belastend was het spelen van het computerspel voor het gezin (u en eventuele andere gezinsleden)?	○	○	○	○	○	○	○	○	○	○

	Te kort									Te lang
	1	2	3	4	5	6	7	8	9	10
3. Wat vindt u van het aantal minuten per dag dat uw kind het computerspel mocht spelen?	○	○	○	○	○	○	○	○	○	○

	Te weinig									Te veel
	1	2	3	4	5	6	7	8	9	10
4. Wat vindt u van het aantal keer dat uw kind het computerspel mocht spelen?	○	○	○	○	○	○	○	○	○	○

	Helemaal niet									Helemaal wel
	1	2	3	4	5	6	7	8	9	10
5. Hoe gemotiveerd was uw kind om het computerspel game te spelen?	○	○	○	○	○	○	○	○	○	○

	Te weinig									Te veel
	1	2	3	4	5	6	7	8	9	10
6. Hoeveel moeite kostte het u om uw kind te motiveren om het computerspel te blijven spelen?	○	○	○	○	○	○	○	○	○	○

	1	2	3	4	5	6	7	8	9	10
7. Op school worden altijd rapportcijfers gegeven, een 10 is zeer goed, een 6 is voldoende en een 1 is zeer slecht. Welk cijfer zou u geven aan het computerspel dat uw kind gespeeld heeft?	○	○	○	○	○	○	○	○	○	○

8. Denkt u dat uw kind iets geleerd heeft van het computerspel?
☐ Ja, omdat…………………………………………………………………………...
☐ Nee, omdat…………………………………………………………………………
☐ Ik weet het niet

Indien ja, beschrijf
……………………………………………………………………………………………………
……………………………………………………………………………………………………
……………………………………………………………………………………………………

9. Heeft u nog op- en/of aanmerkingen met betrekking tot bovengenoemde vragen?
……………………………………………………………………………………………………
……………………………………………………………………………………………………
…………………………………………………………………………………………………..

L. Satisfaction Children, Self constructed questionnaire, version 01

Hallo,

Jij hebt de afgelopen weken meegedaan aan een onderzoek naar een computerspel. Je hebt het computerspel een aantal weken mogen spelen. Wij willen graag jouw mening weten over dit computerspel.

We hebben een aantal vragen, je kan steeds kiezen uit de volgende antwoorden door op het gezichtje te klikken:

Leuk	Gaat wel	Niet leuk
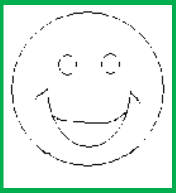	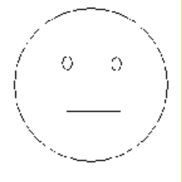	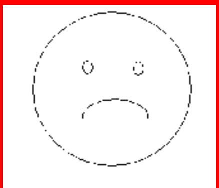

We zijn benieuwd naar jouw mening.

Alvast bedankt voor het invullen.

Hoe vond je het om het computerspel te spelen?	Leuk 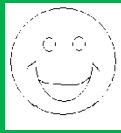	Gaat wel 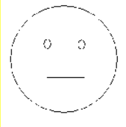	Niet leuk 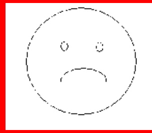
Hoe voelde jij je meestal *voor* het spelen van het computerspel?	Blij 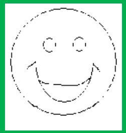	Normaal 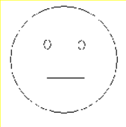	Niet blij 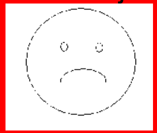
Hoe voelde jij je meestal *na* het spelen van het computerspel?	Blij 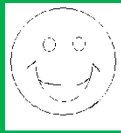	Normaal 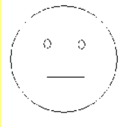	Niet blij 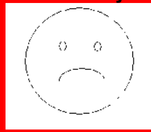
Hoeveel zin had je meestal om het computerspel te spelen?	Veel zin 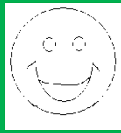	Beetje zin 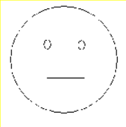	Geen zin 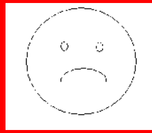
Hoe zou je het vinden om dit computerspel *vaker* te spelen?	Leuk 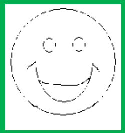	Gaat wel 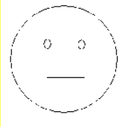	Niet leuk 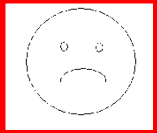

Hoe vind je het dat het spelen van het computerspel afgelopen is?	Jammer<br>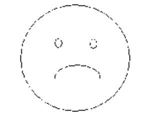	Beetje jammer<br>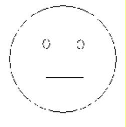	Blij<br>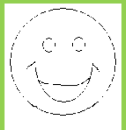
Vind je dat het computerspel jou iets geleerd heeft?	Ja<br>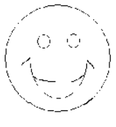	Een beetje<br>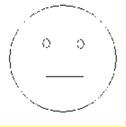	Nee<br>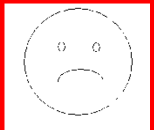
Vind je dat andere kinderen met ADHD ook dit computerspel moeten kunnen spelen?	Ja<br>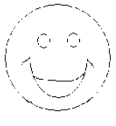	Een beetje<br>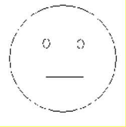	Nee<br>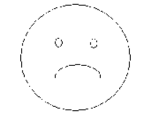
Zou je het leuk vinden om dit computerspel met je vrienden te spelen?	Ja<br>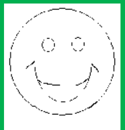	Een beetje<br>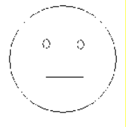	Nee<br>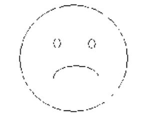

Welk cijfer geef jij het spel?	1	2	3	4	5	6	7	8	9	10

M. Overzicht motivatie

In onderstaand rooster kunt u per speeldag aankruisen in hoeverre u vond dat u uw kind moest motiveren om het computerspel te spelen.

	Niet	Beetje	Veel
Dag 1			
Dag 2			
Dag 3			
Dag 4			
Dag 5			
Dag 6			
Dag 7			
Dag 8			
Dag 9			
Dag 10			
Dag 11			
Dag 12			
Dag 13			
Dag 14			
Dag 15			
Dag 16			
Dag 17			
Dag 18			

Dag 19			
Dag 20			
Dag 21			
Dag 22			
Dag 23			
Dag 24			
Dag 25			
Dag 26			
Dag 27			
Dag 28			
Dag 29			
Dag 30			

Tip: Leg of hang het rooster bij de computer zodat u het niet vergeet in te vullen.
