# Supplementary material for: A serious game for children with Attention Deficit Hyperactivity Disorder: Who benefits the most?
Source: PLoS One. 2018 Mar 15;13(3):e0193681. doi: 10.1371/journal.pone.0193681 (PMC5854282; doi:10.1371/journal.pone.0193681)
Supplement: S3 Fig — (DOCX) [file pone.0193681.s003.docx]

*Appendix 1.* Time management questionnaire.

*Instructions*

Please read the statements below and respond to each one by circling the number (from 1 to 10) which best describes your child during the past two weeks.

False

True

|  | 1 | 2 | 3 | 4 | 5 | 6 | 7 | 8 | 9 | 10 |
| --- | --- | --- | --- | --- | --- | --- | --- | --- | --- | --- |
| 1. My child is able to finish a task within a pre-defined time frame. |  |  |  |  |  |  |  |  |  |  |
| 1. My child can figure out how much time is needed to finish a task before the deadline. |  |  |  |  |  |  |  |  |  |  |
| 1. My child is able to perform the morning routine by himself/herself within a certain time frame. |  |  |  |  |  |  |  |  |  |  |
| 1. My child is able to independently perform daily activities within a reasonable time frame. |  |  |  |  |  |  |  |  |  |  |
| 1. My child is able to adjust his/her homework planning on the basis of other activities (for example: starting earlier to anticipate going to sport or music class that evening) |  |  |  |  |  |  |  |  |  |  |
| 1. My child is able to start his/her long-term projects on time thereby minimizing the chance of time pressure. |  |  |  |  |  |  |  |  |  |  |
| 1. My child often finishes his/her homework before going to bed. |  |  |  |  |  |  |  |  |  |  |
| 1. My child makes good decisions about setting priorities when time is restricted (for example: going home immediately after school to finish a long-term assignment instead of playing with friends). |  |  |  |  |  |  |  |  |  |  |
| 1. My child is able to work on a long-term project over several days. |  |  |  |  |  |  |  |  |  |  |
| 1. My child bungles his/her task at the last moment. |  |  |  |  |  |  |  |  |  |  |
| 1. My child regularly checks his/her watch to keep track of the time. |  |  |  |  |  |  |  |  |  |  |
